# Competition Between Phenothiazines and BH3 Peptide for the Binding Site of the Antiapoptotic BCL-2 Protein

**DOI:** 10.3389/fchem.2020.00235

**Published:** 2020-04-03

**Authors:** Aline Lagoeiro do Carmo, Fernanda Bettanin, Michell Oliveira Almeida, Simone Queiroz Pantaleão, Tiago Rodrigues, Paula Homem-de-Mello, Kathia Maria Honorio

**Affiliations:** ^1^Centro de Ciências Naturais e Humanas, Universidade Federal do ABC, Santo André, Brazil; ^2^Escola de Artes, Ciências e Humanidades, Universidade de São Paulo (USP), São Paulo, Brazil; ^3^Instituto de Química de São Carlos, Universidade de São Paulo (USP), São Paulo, Brazil

**Keywords:** apoptosis, cancer, BCL-2, BH3, phenothiazines, docking, molecular dynamics, binding free energy calculations

## Abstract

The study of proteins and mechanisms involved in the apoptosis and new knowledge about cancer's biology are essential for planning new drugs. Tumor cells develop several strategies to gain proliferative advantages, including molecular alterations to evade from apoptosis. Failures in apoptosis could contribute to cancer pathogenesis, since these defects can cause the accumulation of dividing cells and do not remove genetic variants that have malignant potential. The apoptosis mechanism is composed by proteins that are members of BCL-2 and cysteine-protease families. BH3-only peptides are the “natural” intracellular ligands of BCL-2 family proteins. On the other hand, studies have proved that phenothiazine compounds influence the induction of cellular death. To understand the characteristics of phenothiazines and their effects on tumoral cells and organelles involved in the apoptosis, as well as evaluating their pharmacologic potential, we have carried out computational simulation with the purpose of relating the structures of the phenothiazines with their biological activity. Since the tridimensional (3D) structure of the target protein is known, we have employed the molecular docking approach to study the interactions between compounds and the protein's active site. Hereafter, the molecular dynamics technique was used to verify the temporal evolution of the BCL-2 complexes with phenothiazinic compounds and the BH3 peptide, the stability and the mobility of these molecules in the BCL-2 binding site. From these results, the calculation of binding free energy between the compounds and the biological target was carried out. Thus, it was possible to verify that thioridazine and trifluoperazine tend to increase the stability of the BCL-2 protein and can compete for the binding site with the BH3 peptide.

## Introduction

Apoptosis is a highly regulated form of programmed cell death occurring physiologically in living organisms. However, alterations and defects in this process are also involved in the pathogenesis of several diseases, such as cancer, AIDS, Parkinson and Alzheimer diseases, amyotrophic lateral sclerosis and others (Thompson, 1995). Apoptotic cells exhibit morphological alterations, including plasma membrane blebbing, chromatin condensation, internucleosomal DNA, and formation of apoptotic bodies. Such features result from the action of complex machinery, involving the regulation and execution by BCL-2 family proteins and also by cysteine proteases (initiator or executioner caspases) (Kalkavan and Green, 2018). Particularly regarding cancer, the unlimited proliferative capacity of tumor cells is due to several genetic and molecular alterations, including mechanisms for evading apoptosis (Brown and Attardi, 2005; Hanahan and Weinberg, 2011). One of these mechanisms is the altered expression and function of pro- and antiapoptotic members of B-cell lymphoma-2 (BCL-2) family proteins, directly involved with tumorigenesis and tumor progression/malignance (Coustan-Smith et al., 1996; Gobé et al., 2002). Thus, there are a plenty of molecular studies and clinical trials in course to target BCL-2 proteins to cancer therapy (Adams et al., 2019).

The BCL-2 family is currently divided in proapoptotic members, including BAX and BAK, antiapoptotic members, such as BCL-2, BCL-xL, and MCL-1, and BH3-only proteins (BIM, BID, PUMA, NOXA, and others), which are potent activators of apoptosis (Letai et al., 2002; Youle and Strasser, 2008). Structural characteristics are defined by sequence homology analysis, which allowed identifying four domains (BH1-BH4) involved in protein-protein interactions among members of the BCL-2 family. A hydrophobic slit is formed by BH1, BH2, and BH3 domains, which participates in the uptake of the BH3 domain of pro-apoptotic proteins via heterodimerization; the BH4 domain is present in antiapoptotic activity. The BH3-only proteins just possess the BH3 motif. There are complex interactions among the BCL-2 family members, which comprise a regulatory mechanism of control of cell fate in response to different stimuli. The recruitment of proapoptotic by antiapoptotic proteins occurs through the interaction between the highly conserved helical BH3 domain of the proapoptic protein and a binding groove in the antiapoptotic protein. Considering the homology and structural similarities with the BH3 domain of proapoptotic proteins, BH3-only proteins interact with the binding groove, releasing the proapoptotic proteins and neutralizing the antiapoptotic proteins. In this scenario, BH3-only members have a crucial role in the initiation of apoptotic cell death, since they can bind to the specific domains in anti- or proapoptotic BCL-2 proteins (Lomonosova and Chinnadurai, 2008). It was proposed that BH3-only members can activate directly proapoptotic BAX and BAK. Also, the interaction of BH3-only proteins with antiapoptotic BCL-2 members can disrupts their inhibitory interaction with the proapoptotic members triggering apoptosis (Du et al., 2011; Shamas-Din et al., 2011). As a result, the comprehension of the interaction of BH3-only proteins with other BCL-2 members acting as apoptosis activators resulted in the development of BH3-only mimetic molecules as a strategy to cancer therapy (Merino et al., 2018; Ewald et al., 2019).

**Figure 1:**
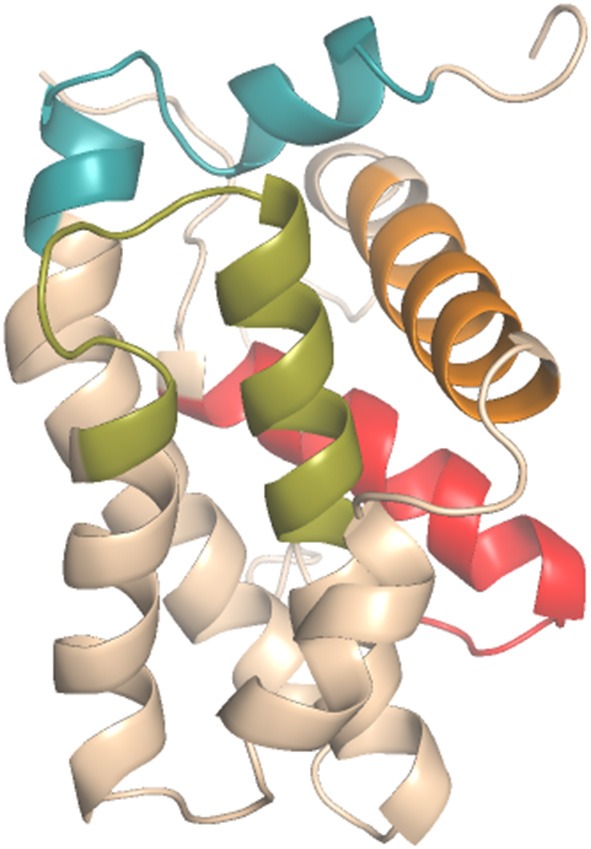
Antiapoptotic BCL-2 protein domains (PDB_ID: 2O22): BH1 (green), BH2 (blue), BH3 (orange), BH4 (red).

Currently, chemicals are under development to inhibit the interactions of pro-apoptotic proteins with the hydrophobic slit of the antiapoptotic protein BCL-2, enabling the imitation of the action of pro-apoptotic proteins with the BH3 domain (Degterev et al., 2001; Delbridge and Strasser, 2015; Zacarías-Lara et al., 2016). Thus, proposals for small molecule interactions with BCL-2 proteins have enabled the development of cancer therapies, including BH3 domain mimetic molecules that bind to the BH3 binding domain in antiapoptotic BCL-2 members such as BCL-2 and BCL-xL (Figure 1 PDB ID: 2O22). As example of these compounds, one may cite ABT-737, navitoclax (ABT-263), obatoclax mesylate (GX15-070), venetoclax (ABT-199), and gossypol and its derivatives (the structures of these compounds are presented in Figures S1A–E) (Oltersdorf et al., 2005; Bajwa et al., 2012; Souers et al., 2013; Pan et al., 2014; Kalkavan and Green, 2018). Thus, several studies are being conducted to identify novel small molecules or peptides able to act as BH3-only mimetics.

**Figure 2:**
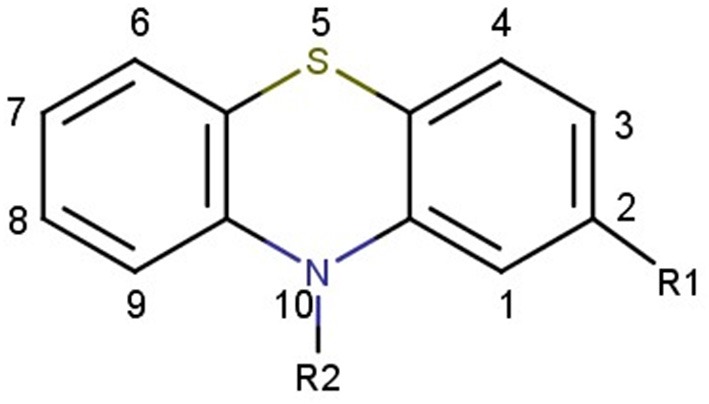
Chemical structure of thiazine nucleus.

In this regards, a class of substances that has potential against BCL-2 refers to phenothiazines (Figure 2), as interactions between these compounds and BCL-2 protein may be favored due to the presence of a polycyclic ring system and different substituents modulating the BCL-2 biological activity. The ability of phenothiazines to interact with hydrophobic slits was previously shown by the interactions of thioridazine with putative binding sites of human thioredoxin 1 (Philot et al., 2016). The interaction between phenothiazines and biological membranes occurs because there is amphipathic character of the molecules. The thiazine nucleus is relatively hydrophobic and its side chain can be hydrophilic and even positively charged depending on the pH of the environment (Homem-de-Mello et al., 2005, 2007; Rodrigues et al., 2006; Perussi, 2007; Rodrigues, 2007; Bettanin et al., 2015; de Faria et al., 2015; Nuñez et al., 2015). Additionally, it has been shown that antipsychotic phenothiazine derivatives possess potent cytotoxicity against several types of tumor cells by triggering of apoptosis, with involvement of mitochondrial permeabilization (de Faria et al., 2015; de Mello et al., 2016; Wu et al., 2016; Chu et al., 2019).

Thus, considering that drug design methods have been employed to understand the interactions between small molecules and biological targets, a study via computational techniques and experimental data of BCL-2 and antipsychotic phenothiazine derivatives was developed applying drug design methods to describe a relationship between chemical structure and biological activity of the selected compounds to propose new drug candidates. Bioinformatics tools were used to characterize possible binding sites and regions for anchoring compounds to the target protein (BCL-2). Molecular docking was also employed to identify the interactions of phenothiazines with the BCL-2 antiapoptotic protein, as well as comparing these results with the interactions of BCL-2 with the BH3 peptide.

## Materials and Methods

**Figure 3:**
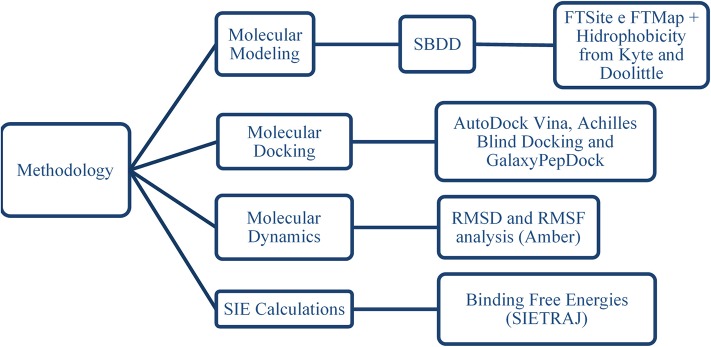
Sequence of methods applied to study the interactions of phenothiazines and the BH3 peptide with BCL-2 protein.

The workflow used to study the interactions between phenothiazines (and the BH3 peptide) and BCL-2 protein is presented in Figure 3.

To study the relationship between chemical structure and biological activity, as well as evaluating the interactions between bioactive ligands and biological targets, molecular modeling tools can be employed to plan new drug candidates (Andricopulo et al., 2009; Sant'Anna, 2009). The strategy employed in this work is known as Structure-Based Drug Design (SBDD), in which three-dimensional biological receptor structures (obtained from experimental techniques such as X-ray diffraction or nuclear magnetic resonance) are used to propose ligand modifications to improve target affinity and specificity (Andricopulo et al., 2009). In this study, the BCL-2 structures obtained from X-ray and nuclear magnetic resonance (NMR), available in the PDB database (PDB_ID: 1YSW, 2O2F, 2O21, 2O22, 2W3L, 4AQ3, 4IEH, 4LVT, 4LXD, 4MAN, 5AGW, 5AGX, 5JSN) were compared to verify significant differences by aligning these structures (Figure S2). The overlap of the structures (Figure S3) was performed in the MUSTANG v3.2.2—Multiple Structural Alignment Algorithm program (Konagurthu et al., 2006).

Given this set of alo-protein structures, multiple alignment was obtained using the Cα atom spatial information with the following steps: (I) calculation of root-mean-square deviation (RMSD) taking into account the distances between the Cα atoms for all structures to detect similar substructures between two structures and obtain a quality value for each possible residue-residue match between the two structures; (II) compute the scores of the corresponding residue-residue pairs; (III) structural alignments in pairs; (IV) recalculation of the scores of the corresponding residue-residue pairs in the context of multiple structures; (V) progressive alignment by using the Mustang algorithm (Konagurthu et al., 2006).

Some sequence failures were observed in all BCL-2 structures, so we have selected the structure obtained via NMR (PDB ID: 2O22) (Bruncko et al., 2007; Rose et al., 2018) because it preserves the loop region. Moreover, besides it is an uncut structure, the backbone alignment is similar to the X-ray structures (Figures S2, S4).

After choosing the more suitable 3D structure, a study was performed to detect the possible binding sites of human BCL-2, followed by the characterization of these regions, using FTSite (Brenke et al., 2009; Ngan et al., 2012; Kozakov et al., 2015) and FTMap (Brenke et al., 2009; Kozakov et al., 2011, 2015; Bohnuud et al., 2012). FTMap is a server that identifies regions in the macromolecule that have important contributions to the ligand-binding free energy (hot spots). For this, the FTMap algorithm uses 16 probe molecules (Table S1) with different shapes, sizes and polarities, which run across the entire surface of the protein looking for the best “positions” for these probes. FTMap is capable of sampling billions of positions for the probe molecules, as well as clustering and ranking them according to an average energy. Consensus sites (CS) are generated, which can be defined as regions at the macromolecule that bind clusters containing different probe molecules, suggesting possible binding hot spots. It is important to highlight that FTMap serves as basis for other algorithms, for example, FTSite that is used to identify ligand binding sites. The main idea of FTSite is ranking the consensus clusters based on the number of non-bonded interactions between the protein and all probe molecules contained in the consensus cluster. So, the amino acid residues interacting with the probe molecules in the top ranked consensus cluster are considered as a possible binding site.

In addition, to better understand the main protein-phenothiazine interactions, the protein hydrophobicity surface was obtained using the UCSF Chimera 1.12 (Pettersen et al., 2004).

**Figure 4:**
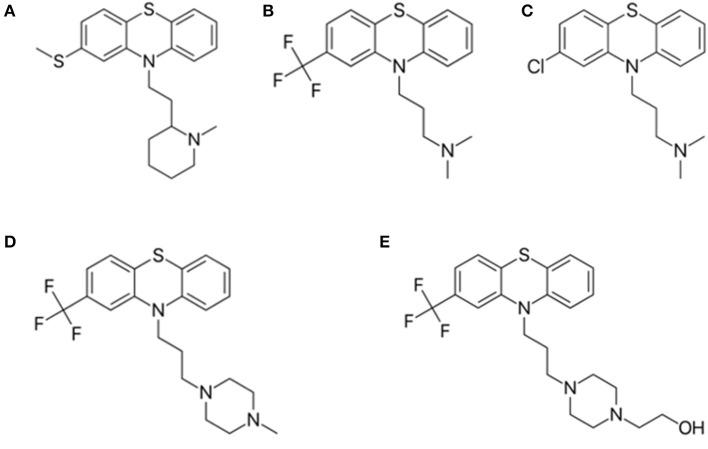
Structure of phenothiazine derivatives analyzed in this study. **(A)** thioridazine; **(B)** triflupromazine; **(C)** chlorpromazine; **(D)** trifluoperazine, and **(E)** fluphenazine.

The phenothiazine derivatives studied here include thioridazine, triflupromazine, chlorpromazine, trifluoperazine, and fluphenazine (Figure 4), which yield relevant *in vitro* cytotoxicity in hepatoma HTC cells (de Faria et al., 2015).

From molecular docking simulations, information on the interaction mode and physicochemical characteristics that affect the affinity of the ligand for the macromolecule is obtained (Wang et al., 2004; Sanchez-Linares et al., 2012). Molecular docking study was performed targeting BCL-2 protein and phenothiazine compounds using the AutoDock Vina 1.5.7. For this, we employed the BCL-2 crystallographic structure (PDB 2O22) with the maximum generation of 10 conformations of each compound. The following parameters were employed in the docking simulations: grid center_x = 4.255, center_y = 1.45, center_z = −5.0, size_x = 25, size_y = 3 and size_z = 34, and exhaustiveness = 20. To validate the docking procedure, redocking analyses were performed in order to recover the original position of the ligand found in the 3D structure of the biological target (Moraes and de Azevedo, 2010).

Visual inspection of the best ligand poses at the target binding site was performed using the PyMOL 2.0, also analyzing the RMSD values calculated by the UCSF Chimera 1.12 and the representation of interactions provided by the Poseview server. It is noteworthy that the RMSD value refers to the average deviation of atoms of an initial structure from the proposed structures and generally the fit is considered successful if the value is below 2.0 Å (Verdonk et al., 2003).

In addition to the AutoDock Vina program, the Achilles Blind Docking server was used to verify molecular interactions in various regions of BCL-2, corroborating the molecular interactions established by phenothiazines in hot spots, where ligands can potentially interact (Brenke et al., 2009; Sanchez-Linares et al., 2012; Kozakov et al., 2015).

For molecular docking and analysis of the interactions between BCL-2 and the BH3 domain, the GalaxyPepDock server was used to analyze protein-protein interactions and better understand cell functions and organization (Lee et al., 2015). In this approach, one of the proteins (or receptor) refers to the origin of the fixed grid coordinate system, and the second protein (or ligand) is defined in a movable grid; interaction energy is defined as a scoring function (Kozakov et al., 2017). To verify the accuracy of the GalaxyPepDock server, redocking analyses with calculation of RMSD values was performed.

The best poses generated by each docking program were selected based on the interactions and binding energies that were generated by the scoring functions, in order to complement the analysis of interactions obtained from the BINANA 1.2.0. This one is able to characterize hydrogen bonding, hydrophobic contact, close contacts, electrostatic interactions, π interactions and salt bridge between receptor—ligand.

After the molecular docking analyses for the five ligands and the BH3 peptide interacting with BCL-2, the next step to be carried out was the preparation of the systems for molecular dynamics (MD) simulations from the calculation of restrained electrostatic potential charges (Wang et al., 2000) of each ligand (from the conformations obtained from molecular docking). For this, we used the Hartree-Fock methodology (Echenique and Alonso, 2007), as implemented in Gaussian09 (Frisch et al., 2009), with 6-31G* basis set (Ditchfield et al., 1971).

Afterwards, the next step related to the preparation of the systems was the solvent box analysis for the target. In this step, we aim to establish the most suitable solvent box for the BCL-2 protein, where the visual analysis was performed in Chimera 1.62 (Pettersen et al., 2004) and the chosen parameters were: periodic octahedral box with a distance of 12 Å between the target and the walls of the box.

From the obtained solvent parameters, the next steps involved in the preparation of the six systems for the MD simulations were: (I) preparation of the topology of the five phenothiazines in the Antechamber module implemented in Ambertools 12 (Salomon-Ferrer et al., 2013) using the RESP charges (charges for BH3 peptide were obtained from the force field); (II) insertion of the FF99SB force field for the coordinates of the six complexes with the Tleap program; (III) total charge calculation of the six systems (the total charge obtained for the six complexes was −9, so 9 sodium ions were inserted to neutralize the system); (IV) inclusion of TIP3P-type water molecules to fill the simulation box; (V) preparation of MD scripts: isothermal-isobaric or NPT ensemble, Langevin thermostat (ntt = 3) and Monte Carlo barostat (Case et al., 2012).

Simulations were then performed following the following steps: (i) four minimizations to eliminate very close contacts between atoms; in the first minimization, the system was kept fixed (without degrees of freedom); in the second and third simulations, only ligands and peptide were kept fixed and in the last minimization, the whole system was free; (ii) heating (thermal bath) from 0 to 300 K, and a time period of 0.5 ns; the purpose of this step was to control the temperature and adjust the kinetic energy of the system; (iii) 10 ns for equilibration of the system, and it is finalized with the thermal equilibrium of the system; (iv) production step to obtain time subtrajectories. In this final step, the system moves freely and, in addition to simulating thermodynamic properties, a lower energy conformation is obtained for each system under study. This last step was performed over a time period of 100 ns; (v) subtrajectory RMSD value analysis (trajectory stability analysis); (vi) analysis of root-mean-square fluctuation (RMSF) values in order to verify the fluctuations that occur between BCL-2 residues in the presence of the ligands.

Finally, SIE (Solvated Interaction Energy) methodology was employed to estimate the binding free energy related to the ligand-receptor complex by applying the boundary element method (BEM) to solve the Poisson-Boltzmann equation. This method also uses implicit solvation in the study of protein-ligand complexes (Naïm et al., 2007; Silva et al., 2016). For this, SIE was used in this study to estimate the binding free energy between BCL-2 and the five phenothiazine derivatives, or the BH3 peptide, from the most stable subtrajectories generated by the MD simulations. Thus, for the SIE method to be implemented, the following steps were performed: (I) solvent removal using the cpptraj program, generating a file without periodic solvation coordinates (SIE uses implicit solvation); (II) elaboration of the file containing the initial and final coordinates of the dynamics trajectory; (III) choice of frame range over start and end frames (each sub-trajectory of molecular dynamics has 500 frames), from frame 1 to 250; (IV) specify the number of atoms of the target, the five ligands and the peptide; (V) output file definition (sie.log), which contains the results obtained; (VI) calculations by the SIETRAJ program and analysis of the estimated free energy values for the six complexes (BCL-2 + phenothiazines and BCL-2 + BH3 peptide).

## Results and Discussion

### Structural Analysis of BCL-2 Structure

**Figure 5:**
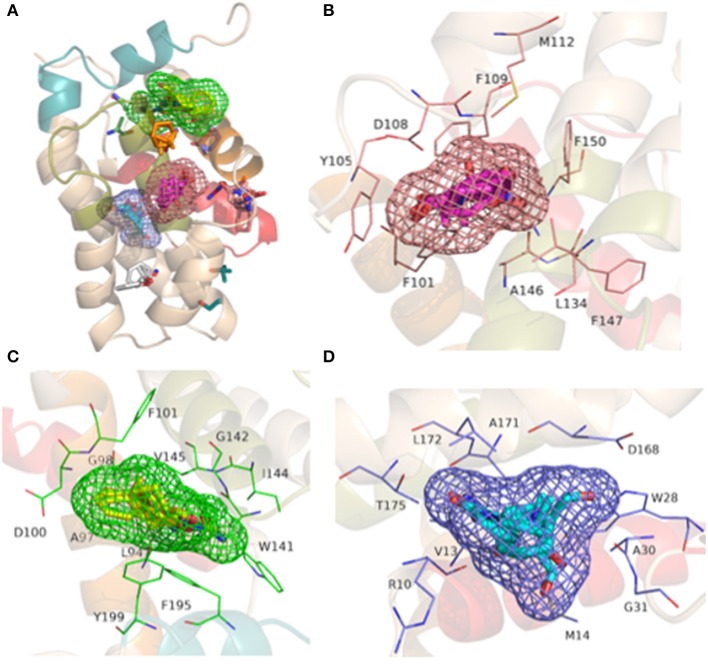
Representation of the BH1-BH4 domains with the presence of the binding sites detected from FTSite and the coupling of probe molecules (in clusters) by the FTMap server produced in the PyMOL 2.0 program: **(A)** the three binding sites with their respective probes; **(B)** Site 1: residues (light pink) with FTSite probes (light pink mesh) and 001 cluster probes (pink); **(C)** Site 2: residues (green) with FTSite probes (green mesh) and 002 cluster probes (yellow) and **(D)** Site 3: residues (blue) with FTSite probes (blue mesh) and 000 cluster probes (light blue).

Three binding site candidates were located on the BCL-2 protein (PDB_ID: 2W3L) using the FTSite and FTMap servers. The detected sites have the following residues: site 1—Phe101, Tyr105, Asp108, Phe109, Met112, Leu134, Ala146, Phe147, Glu149, Phe150; site 2—Leu94, Ala97, Gly98, Asp100, Phe101, Trp141, Gly142, Ile144, Val145, Phe195, Tyr199; e site 3—Arg10, Val13, Met14, Trp28, Ala30, Gly31, Asp168, Ala171, Leu172 e Thr175. Different probe molecules were used to determine which had the highest affinity for each binding site. The results indicate that the three sites have affinity for polar molecules, hydrogen bond donors and acceptors, hydrophobic and aromatic groups. The representation of the detected binding sites containing the clusters of probe molecules according to the affinity of the molecular interaction on the protein is illustrated in Figure 5A. Some probes may also have small contacts with the protein or be in small buried sites, but large CSs occur at the binding site hot spots, also depicted in Figure 5A, where the FTMap probes are not in place connection sites determined by FTSite. Residues of the sites 1, 2, and 3 are also shown in Figures 5B–D, respectively.

**Figure 6:**
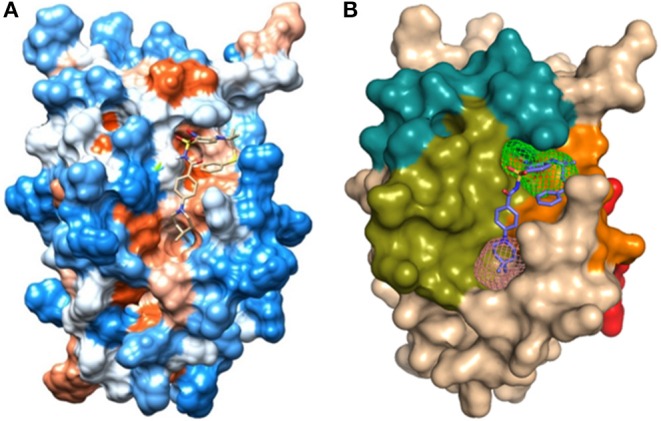
**(A)** Hydrophobicity surface from Kyte and Doolittle (1982) (Table S2): hydrophobic orange-red; neutral white; hydrophilic blue, obtained from UCSF Chimera 1.12 with the crystallographic ligand of protein BCL-2 (PDB_ID: 2O22); **(B)** Representation of the domains: BH1 (green), BH2 (blue), BH3 (orange), BH4 (red), and binding site 1 (pink) and site 2 (green) in the human BCL-2 enzyme - crystallographic structure (PDB_ID: 2O22).

The hydrophobicity scale of the BCL-2 protein was used to complement the results obtained from FTMap and FTSite. Figure 6 indicates that the crystallographic ligand interacts with the sites 1 and 2 due to its structural size and hydrophobic characteristics. In order to understand the position and the interactions of phenothiazines with BCL-2, molecular redocking was required.

Next, the molecular docking procedure was performed with the maximum number of poses to be generated at the defined binding site. Thus, the ligand structure found in the tridimensional structure of BCL-2 was superimposed with the ligand pose obtained via molecular docking (Figure S5). This overlap generated a RMSD value of 1.327 Å, indicating that the parameters used for the redocking analysis reliably reproduce the experimental conformation of the ligand and can be used to dock the phenothiazine derivatives. An analysis of the images generated by the Poseview program shows that the interactions of the ligand (via redocking) with BCL-2 protein are very similar to those observed in the experimental structure (Figure S6).

### Molecular Docking

Once the parameters were validated from the redocking analyses, molecular docking studies between BCL-2 and phenothiazine derivatives were performed. It is noteworthy that, according to the experimental data (de Faria et al., 2015), it is possible to establish the following ascending order for cytotoxicity: chlorpromazine < triflupromazine < fluphenazine < trifluoperazine < thioridazine.

**Figure 7:**
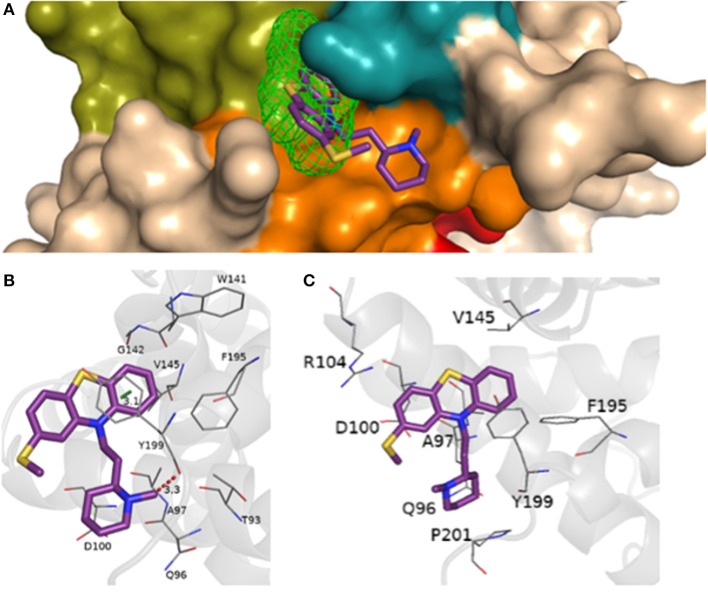
**(A)** Representation of the BH1-BH4 domains with the presence of the site 2 detected from FTSite and the molecular docking of thioridazine, piperidine subclass, EC_50_ = (45.5 ± 1.0) μmol.L^−1^, performed at AutoDock Vina 1.5.7. Representation of hydrogen bonding (red) from the molecular docking performed in AutoDock Vina 1.5.7, **(B)** in the Achilles Blind Docking Server, **(C)** and the additional interactions and/or confirmed by BINANA 1.2.0.

**Figure 8:**
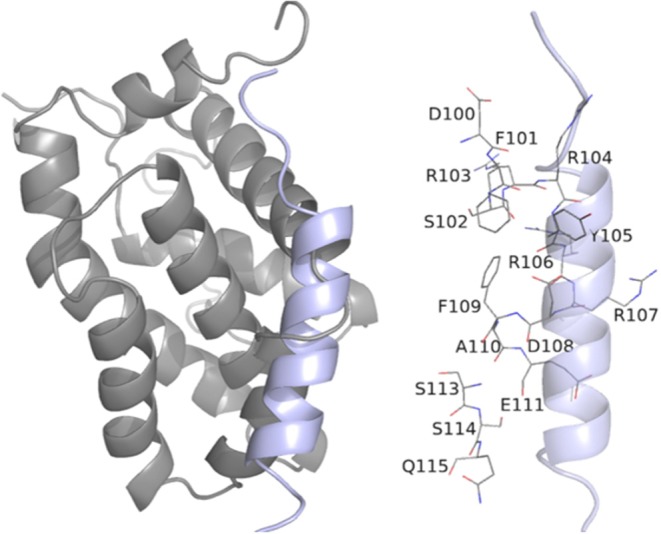
Representation of BCL-2 protein residues with BH3 peptide obtained from molecular docking performed in the GalaxyPepDock server with additional interactions, confirmed by BINANA 1.2.0.

The interaction of the most cytotoxic phenothiazine derivative thioridazine with BCL-2 is depicted in Figure 7 (docking data for the other phenothiazine derivatives are presented in Figures S7–S10). In addition, considering the possible competition between a BH3-only peptide and the thioridazine for BCL-2 binding site, the interaction of BCL-2 and a BH3 domain was evaluated. The docking results considering the interaction between BCL-2 and the BH3 domain obtained from the GalaxyPepDock server are presented in Figure 8. Validation of the docking procedure between BCL-2 and BH3 peptide resulted in a satisfactory RMSD (root-mean-square deviation) value of 1.962 Å and preservation of the alpha helix structure (Figure S11).

From the docking results, it was possible to verify that phenothiazine derivatives showed similar interactions at BCL-2 protein, since we can see that the thiazine nucleus is located at the site 2 recognized by the FTSite server, which has favorable affinities by aromatic and hydrophobic groups.

Both strategies employed to predict the interactions between phenothiazines and BCL-2 are in reasonable agreement with regard to energies and interactions, as can be seen in Tables 1, 2. The ligand-target complexes obtained from the AutoDock Vina program proved to be adequate and confirmed by the Achilles Blind Docking Server, and then, they were used for the molecular dynamics simulations. The main interactions between BCL-2 and the BH3-only peptide are displayed in Table 3, and we can see that the main interactions observed were hydrophobic contacts. This simulation was possible only with the GalaxyPepDock Server, which is specific to identify protein-peptide interactions.

**Table 1:** Description on the interactions between the main residues of BCL-2 and phenothiazines.

**AutoDock Vina 1.5.7**		**Achilles blind docking server**	
**Residue**	**Interaction**	**Residue**	**Interaction**
**Chlorpromazine**			
Ala97	Hydrogen Bond	Ala97	Hydrophobic contact
Asp100	Salt Bridge	Asp100	Salt bridge
Phe101	Hydrophobic contact	Phe101	Hydrophobic contact
Arg104	Hydrophobic contact	Arg104	Hydrophobic contact
Trp141	Hydrophobic contact	Trp141	Hydrophobic contact
Val145	Hydrophobic contact	Val145	Hydrophobic contact
Phe195	π-stacking	Phe195	Hydrophobic contact
		Tyr199	Hydrophobic contact
**Triflupromazine**			
Gln96	Hydrophobic contact	Leu94	Hydrophobic contact
Ala97	Hydrophobic contact	Ala97	Hydrophobic contact
Asp100	Hydrophobic contact	Phe101	Hydrophobic contact
Phe101	Hydrophobic contact	Trp141	Hydrophobic contact
Arg104	Cation-π	Val145	Hydrophobic contact
Val145	Hydrophobic contact	Phe195	Cátion-π
Phe195	Hydrophobic contact	Tyr199	π-stacking
Tyr199	Hydrogen Bond		
Pro201	Hydrophobic contact		
**Fluphenazine**			
Ala97	Hydrophobic contact	Gln96	Hydrophobic contact
Asp100	Hydrophobic contact	Ala97	Hydrophobic contact
Phe101	Hydrophobic contact	Asp100	Hydrophobic contact
Arg104	Cátion-π	Phe101	Hydrophobic contact
Tyr105	Hydrophobic contact	Arg104	Hydrophobic contact
Gly142	Hydrogen Bond	Val145	Hydrophobic contact
Phe195	Hydrophobic contact	Pro201	Hydrophobic contact
Tyr199	π-stacking	Ser202	Hydrogen Bond
**Trifluoperazine**			
Leu94	Hydrogen bond	Thr93	Hydrophobic contact
Ala97	Hydrogen bond	Leu94	Hydrophobic contact
Asp100	Salt bridge	Gln96	Hydrophobic contact
Phe101	Hydrophobic contact	Ala97	Hydrophobic contact
Arg104	Hydrophobic contact	Phe101	Hydrophobic contact
Trp141	Hydrophobic contact	Arg104	Hydrophobic contact
Val145	Hydrophobic contact	Trp141	Hydrophobic contact
Phe195	π-stacking	Val145	Hydrophobic contact
Tyr199	Hydrogen Bond	Phe195	Hydrophobic contact
		Tyr199	Hydrophobic contact
**Thioridazine**			
Thr93	Hydrophobic contact	Gln96	Hydrophobic contact
Gln96	Hydrophobic contact	Ala97	Hydrophobic contact
Ala97	Hydrophobic contact	Asp100	Hydrophobic contact
Asp100	Hydrophobic contact	Arg104	Hydrophobic contact
Trp141	Hydrophobic contact	Val145	Hydrophobic contact
Gly142	Hydrophobic contact	Phe195	Hydrophobic contact
Val145	Hydrophobic contact	Tyr199	Hydrophobic contact
Phe195	Hydrophobic contact	Pro201	Hydrophobic contact
Tyr199	π-stacking and hydrogen Bond		

**Table 2:** EC_50_ values (± 1.0 μmol.L^−1^), interaction energies (kcal.mol^−1^) and number (#) of interactions obtained from AutoDock Vina and Achilles Blind Docking for phenothiazines and BCL-2.

		**Chlorpromazine**	**Triflupromazine**	**Fluphenazine**	**Trifluoperazine**	**Thioridazine**
	${\text{EC}}_{50}^{*}$	125.3	105.9	63.2	56.2	45.5
AutoDock Vina 1.5.7	Interaction energy (kcal.mol^−1^)	−6.4	−6.6	−6.6	−7.1	−6.0
	#hydrophobic contacts	4	7	5	4	8
	#hydrogen bonds	1	1	1	3	1
	# π-stacking	1	0	1	1	1
	#other interactions^**^	1	1	1	1	0
	Total #interactions	7	9	8	9	10
Achilles Blind Docking	Energy (kcal.mol^−1^)	−6.3	−7.1	−7.4	−7.5	−6.8
	#hydrophobic contacts	7	5	7	10	8
	#hydrogen bonds	0	0	1	0	0
	# π-stacking	0	1	0	0	0
	# other interactions^**^	1	1	0	0	0
	Total #interactions	8	7	8	10	8

* *(de Faria et al., 2015)*.

** *salt bridge or cation- π*.

**Table 3:** Description of the interactions between BCL-2 residues and the BH3 peptide.

**GalaxyPepDock Server**	
**Residue**	**Interaction**
Asp100	Hydrophobic contact
Phe101	Hydrophobic contact
Ser102	Hydrophobic contact
Arg103	Hydrophobic contact
Arg104	Hydrophobic contact
Tyr105	Hydrophobic contact
Arg106	Hydrophobic contact
Arg107	Hydrogen Bond and Salt Bridge
Asp108	Hydrophobic contact
Phe109	Hydrophobic contact
Ala110	Hydrophobic contact
Glu111	Hydrophobic contact
Met112	Hydrophobic contact
Ser114	Hydrophobic contact
Gln115	Hydrophobic contact

Through the analysis of the bioactive conformations obtained from both programs, it was observed that the thiazine nucleus present in the studied ligands showed affinity by BCL-2, possibly due to the presence of aromatic and hydrophobic groups, which was also suggested by the FTSite server. In order to understand dynamic and energetic factors involved in the interaction between the phenothiazines and BCL-2, molecular dynamics simulations and calculations of binding free energy (ΔG) were performed.

### Molecular Dynamics

**Figure 9:**
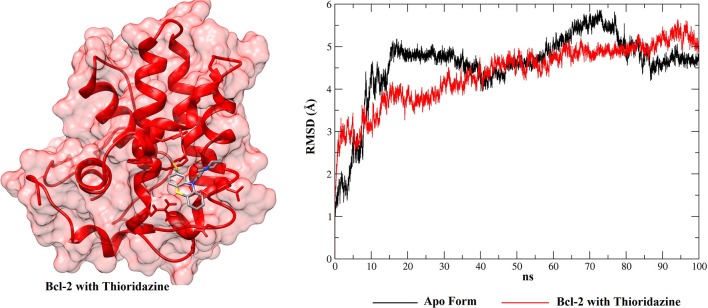
RMSD values of BCL-2 in the apo form (black), BCL-2 with thioridazine (red) and 3D conformation obtained from the clustering of all structures obtained from the MD simulation.

**Figure 10:**
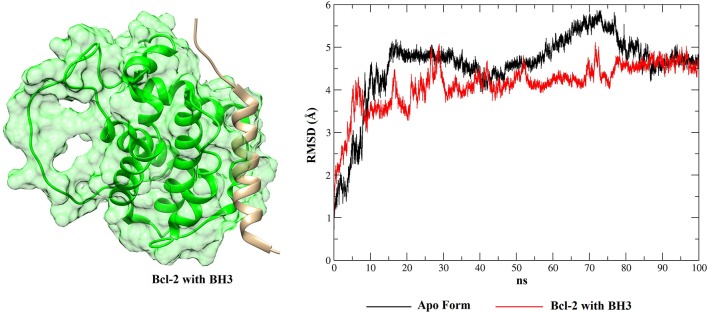
RMSD values of BCL-2 in the apo form (black), BCL-2 with the BH3 peptide (red) and 3D conformation obtained from the clustering of all structures obtained from the MD simulation.

From the molecular docking between phenothiazines and BCL-2 (and between the peptide BH3 and BCL-2), the possible conformations of the phenothiazine derivatives and, additionally the BH3 peptide to evaluate competition, were chosen to perform MD simulations. Then, after the end of the simulations, the RMSD plots were generated from the Cpptraj platform (Amber 12). This analysis was performed to verify the temporal evolution of the complexes BCL-2 + phenothiazines and BCL-2 + BH3, as well as the stability and the mobility of the formed systems. MD simulations were also performed with the apo BCL-2 target (no ligand at its binding site) and, in addition to the RMSD plots, the structures observed along 100 ns of MD simulations were clustered for all complexes and the protein in the form apo using Chimera 1.13.1. Figures 9, 10 show the RMSD plots for the complexes formed by BCL-2 + thioridazine and BCL-2 + BH3. The results obtained from the MD simulations for the other phenothiazines are presented in Figures S12–S15.

From the RMSD plots for the BCL-2 trajectories, the five phenothiazine compounds and the BH3 peptide, an analysis of trajectory stability and mobility can be performed. Thus, Figure 9 shows that thioridazine increases the level of stability of the complex, because BCL-2 with this molecule coupled at its binding site exhibits lower RMSD variations. The second complex with the smallest variations is shown in Figure S12 (Supplementary Material—BCL-2 + trifluoperazine). These two phenothiazine compounds have the lowest EC_50_ values for cytotoxicity against HTC cells (45.5 ± 1.0 and 56.2 ± 1.0 μmol.L^−1^, respectively) and these results showed that BCL-2 has greater stability in the presence of these two ligands. Compared to other phenothiazine compounds (Figures S13–S15), RMSD results show greater variations, i.e., suggesting that fluphenazine, trifluopromazine and chlorpromazine decreased the target stability.

With respect to the peptide BH3, Figure 10 shows that the receptor exhibits smaller RMSD variations than for the phenothiazines, suggesting that the peptide BH3 assists in increasing the target stability. However, Figure 10 also shows that BH3 is the ligand that exhibits greater variations in RMSD and this can be explained by the size of this peptide relative to the phenothiazine compounds.

**Figure 11:**
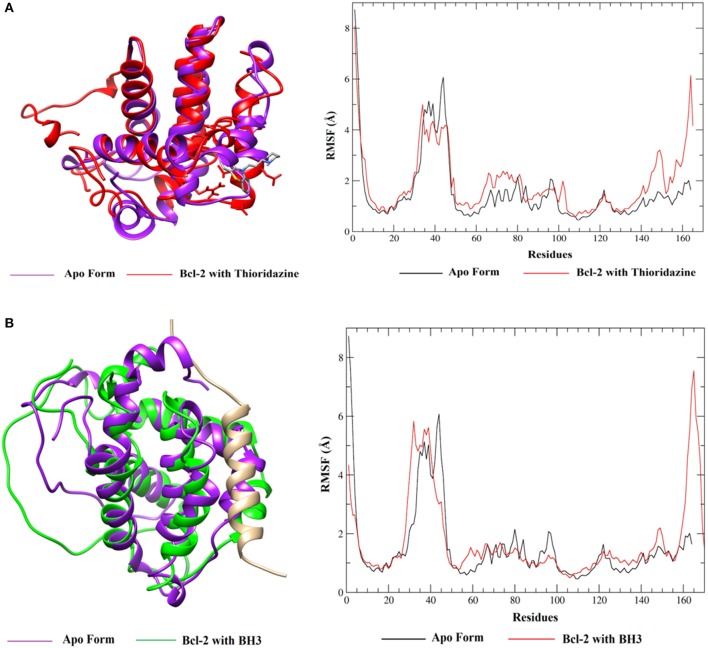
RMSF values of BCL-2 in the presence of **(A)** thioridazine and **(B)** BH3-only peptide.

When these RMSD values are compared with the apo form, thioridazine and the BH3 peptide cause structural changes in the BCL-2 target; however, when this receptor has the thioridazine molecule in its binding site, the complex is more stable. It is also noted that in all cases the RMSD variations for the receptors are >4 Å and this can be explained by loops on the BCL-2 chains, which increase their mobility. These results are corroborated by the RMSF plots, which are displayed in Figure 11 for thioridazine and the BH3 peptide, respectively. RMSF plots for the other phenothiazines can be viewed in Figures S16–S19.

RMSF plots were generated in order to verify the flexibility and the mobility of the backbone atoms at the complexes. The plots show the different amplitudes of fluctuations in the apo and holo forms (Figure 11A and Figure S16) and indicate that the complexes with thioridazine and trifluoperazine have the smallest fluctuations in the main residues, relative to the other phenothiazine derivatives. It is also noted that there is a large fluctuation of residues in the loop regions. For the BH3 peptide (Figure 11B), the fluctuations of the amino acid residues are smaller, confirming the RMSD values. Some loop regions of both BCL-2 and the peptide structure show higher fluctuations, suggesting that the peptide is more mobile compared to other compounds because it has a larger number of atoms in its structure and has side chains and a loop.

Therefore, from the results obtained from MD simulations (RMSD and RSMF values) it was possible to verify that thioridazine and trifluoperazine tend to increase the stability of the BCL-2 protein. From Figure 11, we can also verify the alignment of the conformations generated after the clustering, the differences in the fluctuations of the apo-BCL-2 residues and the complexes (BCL-2 + ligands). Thus, from these results, the next step of this work involved the calculation of the binding free energy between BCL-2 and the phenothiazine derivatives or the BH3-only peptide using the SIE method.

### Free Energy Calculation via SIE Method

After choosing the sub-trajectories that presented the smallest variations (from RMSD values derived from MD simulations), it was possible to estimate the binding free energy (ΔG) of BCL-2 and the molecules under study, using the SIE method. These simulations were performed in order to analyze the stability of BCL-2 in relation to the six ligands. Thus, the ΔG values calculated by the SIE method are presented in Table 4.

**Table 4:** Half maximal effective concentration (EC50) number of interactions obtained with the different softwares (Total #interactions), binding free energy (ΔG) and binding free energy divided by the number of interactions (ΔG/#interactions) calculated for the six complexes (BCL-2 + phenothiazines and BCL-2 + BH3).

**Ligand**	EC_**50**_ (± 1.0 μmol.L^**−1**^)^*^	**Total #interactions (Vina/Achilles)**	**ΔG (kcal.mol^**−1**^)**	**ΔG/#interactions (kcal.mol**^****−1****^**)**	
				**Vina**	**Achilles**
Thioridazine	45.5	10/8	−7.00	−0.70	−0.88
Trifluoperazine	56.2	9/10	−6.47	−0.72	−0.65
Fluphenazine	63.2	8/8	−5.51	−0.69	−0.69
Triflupromazine	105.69	9/7	−4.93	−0.55	−0.70
Chlorpromazine	125.3	7/8	−4.05	−0.58	−0.51
BH3	-	16^**^	−12.81	−0.80	

* *de Faria et al. (2015)*.

** *Obtained from Galaxy PepDock Server*.

The EC_50_ values of phenothiazines were directly proportional to the binding ΔG values. The interaction of BCL-2 with thioridazine has a ΔG^SIE^ value of −7.00 kcal.mol^−1^, while chlorpromazine (the highest EC_50_ value) has the lowest binding free energy value when interacts with BCL-2 (−4.05 kcal.mol^−1^). As expected from its physiological role, the ΔG value for the BH3 peptide was the highest (−12.81 kcal.mol^−1^) that corroborates the RMSD/RSMF analysis and reinforces that BCL-2 presents a strong interaction with the peptide, increasing its stability.

The results obtained from the SIE method also showed that thioridazine and trifluoperazine could inhibit the target BCL-2, just like BH3 peptide, because complexes are favorable and stable since its ΔG values are the most negative. Since most of the interactions between ligands and protein are hydrophobic, one can estimate the free energy of interaction per site (ΔG/#interactions in Table 4) by combining SIE free energies and the number of interactions obtained from docking. This analysis is proposed here since the molecules are smaller than the peptide, and so are the number of possible interactions. Then, if one ligand can interact in the same intensity per site of interaction as BH3 peptide, this ligand can compete for the binding site. Of course, this is a simplistic analysis because each interaction may be stronger or weaker, but it is insightful to verify that thioridazine and trifluoperazine have interaction energy per binding site comparable to BH3 peptide.

## Conclusions

From this study on the BCL-2 protein (involved in the apoptosis process) and some phenothiazine derivatives that have pharmacological properties, we can concluded that phenothiazines may compete with pro-apoptotic proteins. These results were obtained from molecular docking, RMSD and RMSF values and binding free energy.

Docking simulations were important to understand the main interactions between the target (BCL-2) and the phenothiazine compounds. RMSD results for the complex formed between BCL-2 and the two most active phenothiazine compounds (thioridazine and trifluoperazine) suggest that BCL-2 has a higher stability in the presence of these two ligands. Compared to other phenothiazine compounds, RMSD results show greater variations, i.e., the results suggest that fluphenazine, triflupromazine and chlorpromazine decrease the target stability. RMSF plots for the trajectories of BCL-2 and the five phenothiazine compounds showed that thioridazine and trifluoperazine have the smallest fluctuations considering the major residues compared to other phenothiazine compounds.

The binding free energy between BCL-2, the phenothiazine compounds and the BH3 peptide was calculated using the SIE method and the results obtained indicated that the phenothiazine compounds with lower EC_50_ values presented greater affinity (measured by means of ΔG). The net binding energy for BH3 peptide is larger than the net binding energy obtained for the phenothiazines, since BH3 is a larger compound, with many different points for interaction with BCL-2. Moreover, BH3 is the natural ligand of BCL-2, selected evolutionarily to bind. However, our data indicate that the interactions are quite specific for the compounds with greater EC_50_; this interaction can generate competition in specific situations, including chemotherapy in tumor cells, which would induce cell death, and can act as co-adjuvants by this mechanism. Thus, the results obtained in this study can help to better understand the mechanisms involved in the interaction of BCL-2 and phenothiazine compounds and, consequently, may help the design of new substances with improved activity against BCL-2. It should be noted that the inhibition of the antiapoptotic protein BCL-2 by phenothiazines may help explain its apoptosis-inducing effect on tumor cells.

## Data Availability Statement

All datasets generated for this study are included in the article/Supplementary Material.

## Author Contributions

AC and SP carried out calculations (characterization of the protein and docking). MO performed molecular dynamics and SIE calculations, TR was the experimental collaborator (proposed the problem and obtained EC50 data). FB participated on discussions and writing the manuscript. PH-d-M and KH proposed and supervised all the computational simulations, organized discussions with experimental collaborator and wrote/revised the manuscript.

### Conflict of Interest

The authors declare that the research was conducted in the absence of any commercial or financial relationships that could be construed as a potential conflict of interest.
