# Supplementary material for: Competition Between Phenothiazines and BH3 Peptide for the Binding Site of the Antiapoptotic BCL-2 Protein
Source: Front Chem. 2020 Apr 3;8:235. doi: 10.3389/fchem.2020.00235 (PMC7145989; doi:10.3389/fchem.2020.00235)
Supplement: Supplementary file 1 [file Presentation_1.pdf]

Supplementary Material

Competition between phenothiazines and BH3 peptide for the binding site of the antiapoptotic BCL-2 protein

Aline Lagoeiro do Carmo[a], Fernanda Bettanin[b], Michell de Oliveira Almeida[c], Simone Queiroz Pantaleão[a], Tiago Rodrigues[a], Paula Homem-de-Mello[a,*], Kathia Maria Honorio[a,b,*]

[a] Centro de Ciências Naturais e Humanas, Universidade Federal do ABC, Santo André, São Paulo, Brazil.
[b] Escola de Artes, Ciências e Humanidades, Universidade de São Paulo (USP), São Paulo, Brazil.
[c] Instituto de Química de São Carlos, Universidade de São Paulo (USP), São Carlos, São Paulo, Brazil.

*Corresponding author emails: paula.mello@ufabc.edu.br, kmhonorio@usp.br

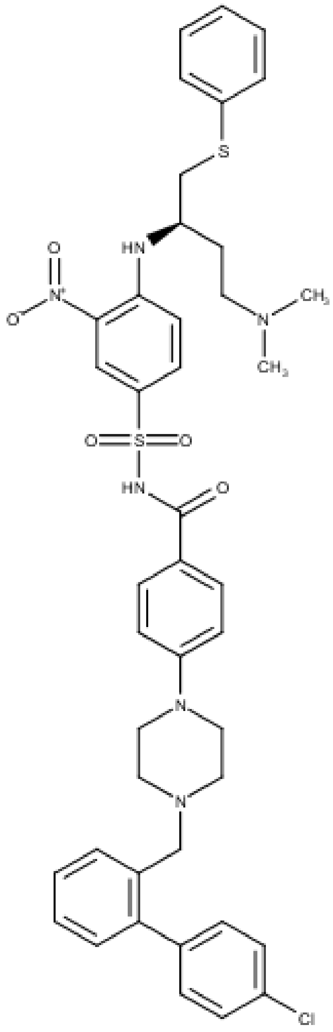

(a)

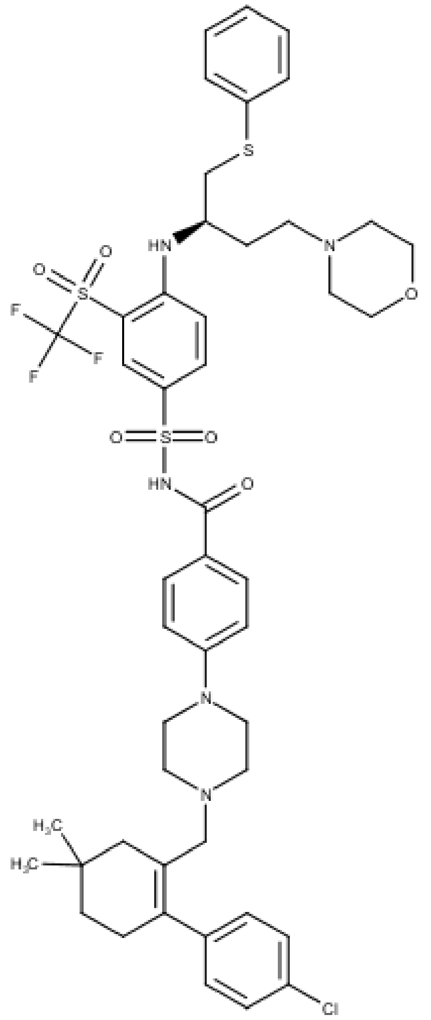

(b)

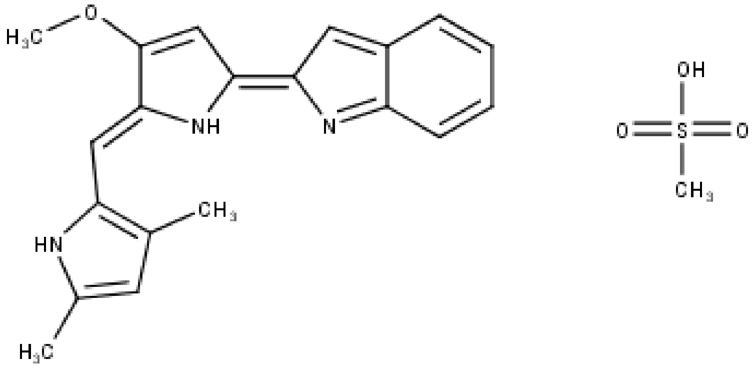

(c)

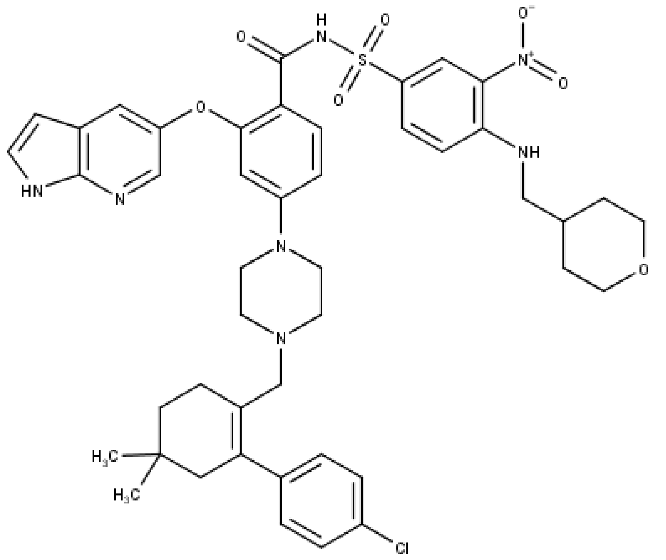

(d)

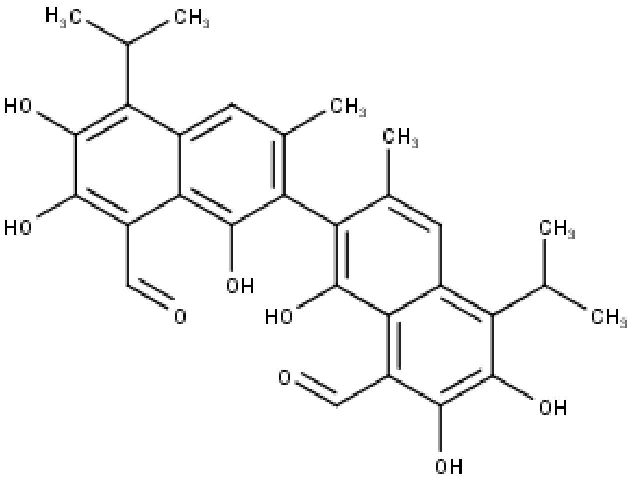

(e)

Figure S1. Structures of the compounds which are intended to mimic the BH3 domain that bind to the BH3 binding domain on BCL-2 antiapoptotic members: (a) ABT-737, (b) navitoclax (ABT-263), (c) obatoclax mesylate (GX15-070), (d) venetoclax (ABT-199) and (e) gossypol.

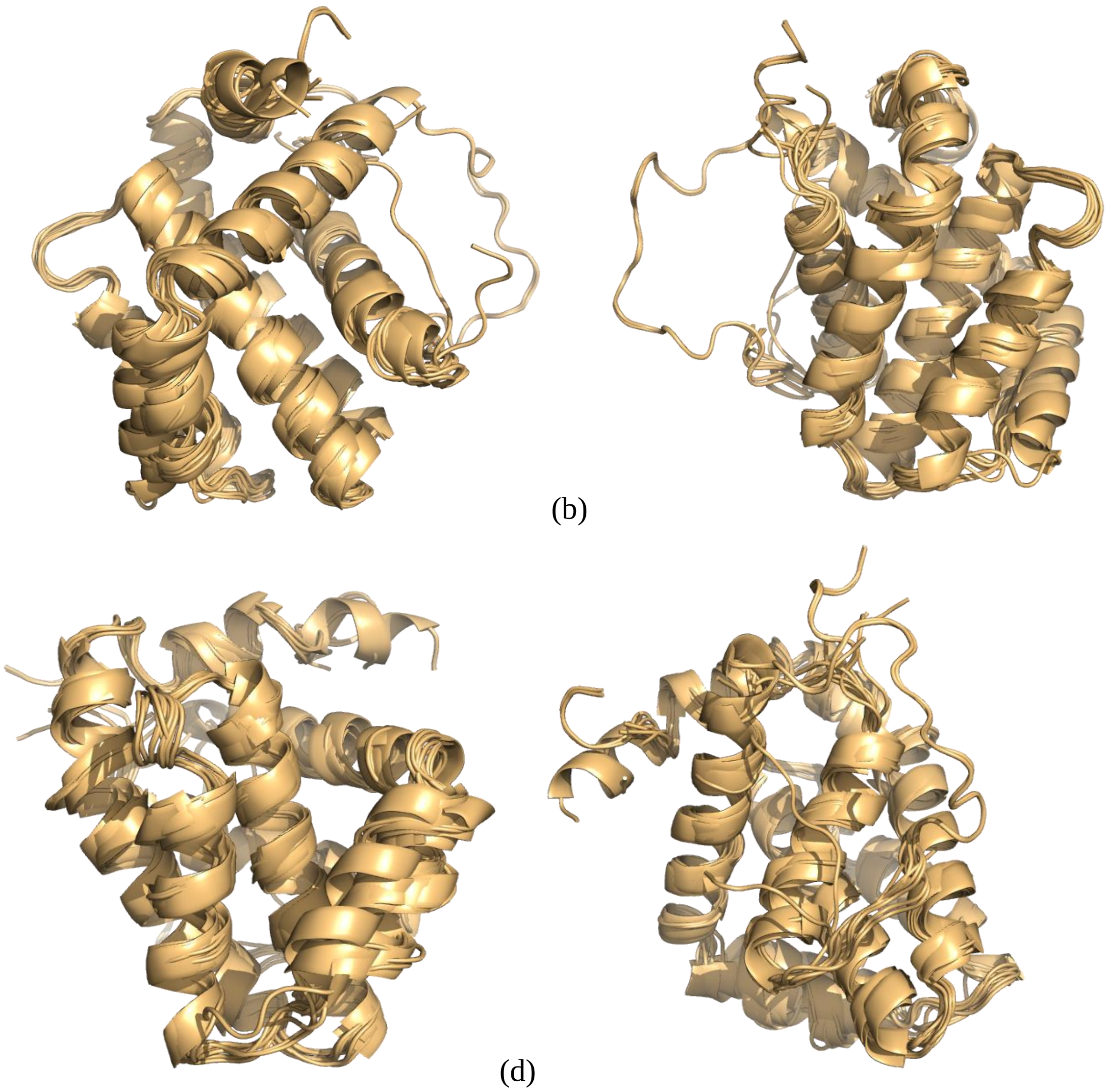

(a) (b) (c) (d)

Figure S2. Alignment of human Bcl-2 protein A chain crystallographic structures (PDB_ID: 1YSW, 2O2F, 2O21, 2O22, 2W3L, 4AQ3, 4IEH, 4LVT, 4LXD, 4MAN, 5AGW, 5AGX, 5JSN): (a) left side view; (b) right side view, (c) front view and (d) rear view.

	1 (1YSW)	2 (2O2F)	3 (2O21)	4 (2O22)	5 (2W3L)	6 (4AQ3)	7 (4IEH)	8 (4LVT)	9 (4LXD)	10 (4MAN)	11 (5AGW)	12 (5AGX)	13 (5JSN)
1	---	1.6	0.0	0.5	1.3	1.3	1.3	1.2	1.3	1.3	1.4	1.4	1.2
2	1.6	---	1.6	1.6	1.6	1.5	1.4	1.4	1.4	1.4	1.6	1.6	1.7
3	0.0	1.6	---	0.5	1.3	1.3	1.3	1.2	1.3	1.3	1.4	1.4	1.2
4	0.5	1.6	0.5	---	1.4	1.3	1.3	1.2	1.3	1.3	1.4	1.5	1.3
5	1.3	1.6	1.3	1.4	---	0.7	1.0	0.7	0.9	0.9	0.8	0.8	0.7
6	1.3	1.5	1.3	1.3	0.7	---	0.8	0.9	0.7	0.8	0.8	0.9	0.8
7	1.3	1.4	1.3	1.3	1.0	0.8	---	0.9	0.5	0.5	0.8	0.9	1.0
8	1.2	1.4	1.2	1.2	0.7	0.9	0.9	---	0.8	0.8	0.9	1.1	0.9
9	1.3	1.4	1.3	1.3	0.9	0.7	0.5	0.8	---	0.5	0.8	0.9	0.9
10	1.3	1.4	1.3	1.3	0.9	0.8	0.5	0.8	0.5	---	0.9	0.9	1.0
11	1.4	1.6	1.4	1.4	0.8	0.8	0.8	0.9	0.8	0.9	---	0.6	0.7
12	1.4	1.6	1.4	1.5	0.8	0.9	0.9	1.1	0.9	0.9	0.6	---	0.9
13	1.2	1.7	1.2	1.3	0.7	0.8	1.0	0.9	0.9	1.0	0.7	0.9	---

Figure S3. RMSD matrix of the alignment of the human Bcl-2 protein A chain crystallographic structures (PDB_ID: 1YSW, 2O2F, 2O21, 2O22, 2W3L, 4AQ3, 4IEH, 4LVT, 4LXD, 4MAN, 5AGW, 5AGX, 5JSN).

```
1ysw.pdb     1   -HAGRTGYDNREIVMKYIHYKLSQRGYEWD--A---GDDVEENRTEAPEGTESEVVHLTL   54
2o2f.pdb     1   ------GYDNREIVMKYIHYKLSQRGYEWD----------------------E-VVHLTL   31
2o21.pdb     1   -HAGRTGYDNREIVMKYIHYKLSQRGYEWD--A---GDDVEENRTEAPEGTESEVVHLTL   54
2o22.pdb     1   -HAGRTGYDNREIVMKYIHYKLSQRGYEWD--A---GDDVEENRTEAPEGTESEVVHLTL   54
2w3l.pdb     1   -------YDNREIVMKYIHYKLSQRGYEWD--A-------------------SEVVHKTL   32
4aq3.pdb     1   -------YDNREIVMKYIHYKLSQRGYEWD------------------------VVHLAL   29
4ieh.pdb     1   -------YDNREIVMKYIHYKLSQRGYEWD----------------------SEVVHLTL   31
4lvt.pdb     1   -------YDNREIVMKYIHYKLSQRGYEWD--A-------------------SEVVHLTL   32
4lxd.pdb     1   R----TGYDNREIVMKYIHYKLSQRGYEWDAG--------------------SEVVHLTL   36
4man.pdb     1   ------GYDNREIVMKYIHYKLSQRGYE-------------------------EVVHLTL   29
5agw.pdb     1   ------GYDNREIVMKYIHYKLSQRGYEWD----------------------SEVVHLTL   32
5agx.pdb     1   ------GYDNREIVMKYIHYKLSQRGYEWD--A-------------------SEVVHLTL   33
5jsn.pdb     1   -----TGYDNREIVMKYIHYKLSQRGYEWD--ASPV----------------PPVVHLTL   37
                        YDNREIVMKYIHYKLSQRGYE                          VVHltL

1ysw.pdb    55   RQAGDDFSRRYRRDFAEMSSQLH----LTPFTARGRFATVVEELFRDGVNWGRIVAFFEF  110
2o2f.pdb    32   RQAGDDFSRRYRRDFAEMSSQLHLTPF----TARGRFATVVEELFRDGVNWGRIVAFFEF   87
2o21.pdb    55   RQAGDDFSRRYRRDFAEMSSQLH----LTPFTARGRFATVVEELFRDGVNWGRIVAFFEF  110
2o22.pdb    55   RQAGDDFSRRYRRDFAEMSSQLH----LTPFTARGRFATVVEELFRDGVNWGRIVAFFEF  110
2w3l.pdb    33   REAGDDFSRRYRRDFAEMSSGLH----LTPFTARGRFATVVEELFRDGVNWGRIVAFFEF   88
4aq3.pdb    30   RQAGDDFSRRYRGDFAEMSSQLH----LTPFTARGRFATVVEELFRDGVNWGRIVAFFEF   85
4ieh.pdb    32   RQAGDDFSRRYRRDFAEMSSQLH----LTPFTARGRFATVVEELFRDGVNWGRIVAFFEF   87
4lvt.pdb    33   RQAGDDFSRRYRRDFAEMSSQLH----LTPFTARGRFATVVEELFRDGVNWGRIVAFFEF   88
4lxd.pdb    37   RQAGDDFSRRYRRDFAEMSSQLH----LTPFTARGRFATVVEELFRDGVNWGRIVAFFEF   92
4man.pdb    30   RQAGDDFSRRYRRDFAEMSSQLH----LTPFTARGRFATVVEELFRDGVNWGRIVAFFEF   85
5agw.pdb    33   RQAGDDFSRRYRRDFAEMSSQLH----LTPFTARGRFATVVEELFRDGVNWGRIVAFFEF   88
5agx.pdb    34   RQAGDDFSRRYRRDFAEMSSQLH----LTPFTARGRFATVVEELFRDGVNWGRIVAFFEF   89
5jsn.pdb    38   RQAGDDFSRRYRRDFAEMSSQLH----LTPFTARGRFATVVEELFRDGVNWGRIVAFFEF   93
                 RqAGDDFSRRYRrDFAEMSSqLH        TARGRFATVVEELFRDGVNWGRIVAFFEF

1ysw.pdb   111   GGVMCVESVNREMSPLVDNIALWMTEYLNRHLHTWIQDNGGWDAFVELYGPSMR--  164
2o2f.pdb    88   GGVMCVESVNREMSPLVDNIALWMTEYLNRHLHTWIQDNGGWDAFVELYGP-----  138
2o21.pdb   111   GGVMCVESVNREMSPLVDNIALWMTEYLNRHLHTWIQDNGGWDAFVELYGPSMR--  164
2o22.pdb   111   GGVMCVESVNREMSPLVDNIALWMTEYLNRHLHTWIQDNGGWDAFVELYGPSMR--  164
2w3l.pdb    89   GGVMCVESVNREMSPLVDNIALWMTEYLNRHLHTWIQDNGGWDAFVELYGPSM---  141
4aq3.pdb    86   GGVMCVESVNREMSPLVDNIALWMTEYLNRHLHTWIQDNGGWDAFVELYG------  135
4ieh.pdb    88   GGVMCVESVNREMSPLVDNIALWMTEYLNRHLHTWIQDNGGWDAFVELYGP-----  138
4lvt.pdb    89   GGVMCVESVNREMSPLVDNIALWMTEYLNRHLHTWIQDNGGWDAFVELYGP-----  139
4lxd.pdb    93   GGVMCVESVNREMSPLVDNIALWMTEYLNRHLHTWIQDNGGWDAFVELYGP-----  143
4man.pdb    86   GGVMCVESVNREMSPLVDNIALWMTEYLNRHLHTWIQDNGGWDAFVELYGP-----  136
5agw.pdb    89   GGVMCVESVNREMSPLVDNIALWMTEYLNRHLHTWIQDNGGWDAFVELYGP-----  139
5agx.pdb    90   GGVMCVESVNREMSPLVDNIALWMTEYLNRHLHTWIQDNGGWDAFVELYGP-----  140
5jsn.pdb    94   GGVMCVESVNREMSPLVDNIALWMTEYLNRHLHTWIQDNGGWDAFVELYGPSMRLE  149
                 GGVMCVESVNREMSPLVDNIALWMTEYLNRHLHTWIQDNGGWDAFVELYG
```

Figure S4. Alignment of A-chain crystallographic structures of human Bcl-2 protein (PDB_ID: 1YSW, 2O2F, 2O21, 2O22, 2W3L, 4AQ3, 4IEH, 4LVT, 4LXD, 4MAN, 5AGW, 5AGX, 5JSN), representing hydrophobic residues including aromatics (red), acids (blue), basic (pink) and basic with hydroxyl groups and / or amino groups (green); with the marking line below each stretch of the multiple alignment indicating fully conserved residues (upper case) and partially conserved residues (lower case).

Table S1. Characteristics of probes used by FTSite and FTMap servers

**Probe**	**Properties**[a]
Acetamide (ACD)	Polar, hydrogen bond acceptor and donor
Acetonitrile (ACN)	Polar and hydrogen bond acceptor character
Acetone (ACT)	Polar and hydrogen bond acceptor character
Acetaldehyde (ADY)	Polar and hydrogen bond acceptor character
Methylamine (AMN)	Polar, hydrogen bond acceptor and donor
Benzaldehyde (BDY)	Polar, aromatic and hydrogen bond acceptor character
Benzene (BEN)	Hydrophobic and aromatic
Butanol (BUT)	Polar and hydrogen bond acceptor character
Ciclohexane (CHX)	Polar, hydrogen bond acceptor and donor
N,N-dimethylformamide (DFO)	Polar and hydrogen bond acceptor character
Dimethyl ether (DME)	Polar and hydrogen bond acceptor character
Ethanol (EOL)	Polar, hydrogen bond acceptor and donor
Ethane (ETH)	Hydrophobic
Phenol (PHN)	Polar, aromatic, hydrogen bond acceptor and donor
Isopropanol (THS)	Polar and hydrogen bond acceptor character
Urea (URE)	Polar, hydrogen bond acceptor and donor

(a) (Bohnuud et al. 2012; Brenke et al. 2009; D. Kozakov et al. 2011; Dima Kozakov et al. 2015; Ngan et al. 2012)

Table S2. Hydrophobicity and hydrophilicity values by Kyte and Doolittle

**Residues**	**Kyte-Doolittle Values** [a]	**Classifications** [b]	**Color Scale** [c]
Ile	4.5	Hydrophobic	Orange-red
Val	4.2	Hydrophobic	Orange-red
Leu	3.8	Hydrophobic	Orange-red
Phe	2.8	Hydrophobic	Orange-red
Cys	2.5	Hydrophobic	Orange-red
Met	1.9	Hydrophobic	Orange-red
Ala	1.8	Hydrophobic	Orange-red
Gly	-0.4	Neutral	White
Thr	-0.7	Neutral	White
Ser	-0.8	Neutral	White
Trp	-0.9	Neutral	White
Tyr	-1.3	Neutral	White
Pro	-1.6	Neutral	White
His	-3.2	Hydrophilic	Blue
Glu	-3.5	Hydrophilic	Blue
Gln	-3.5	Hydrophilic	Blue
Asp	-3.5	Hydrophilic	Blue
Asn	-3.5	Hydrophilic	Blue
Lys	-3.9	Hydrophilic	Blue
Arg	-4.5	Hydrophilic	Blue

(a) Kyte and Doolittle, 1982.

(b) De Oliveira Rodrigues et al., 2015.

(c) Pettersen et al., 2004.

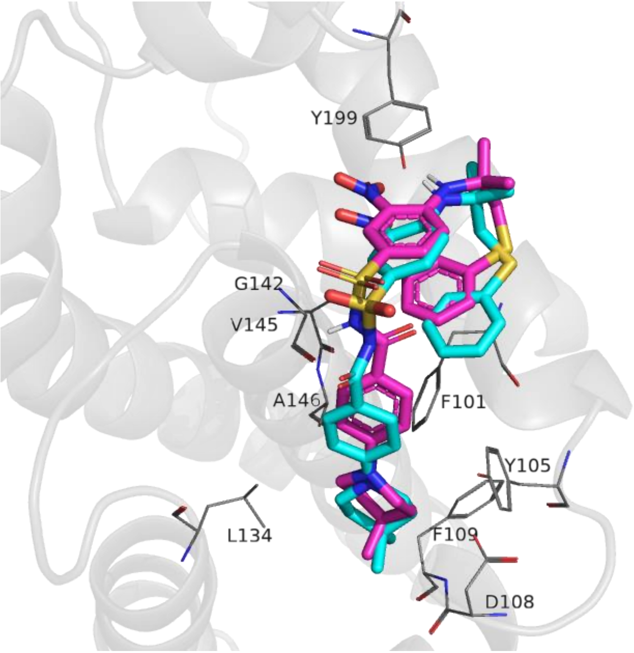

Figure S5. Representation of the molecular coupling of the ligand at the original position of the protein-ligand complex crystal structure (light pink) (PDB_ID: 2O22) and ligand overlap (Blue).

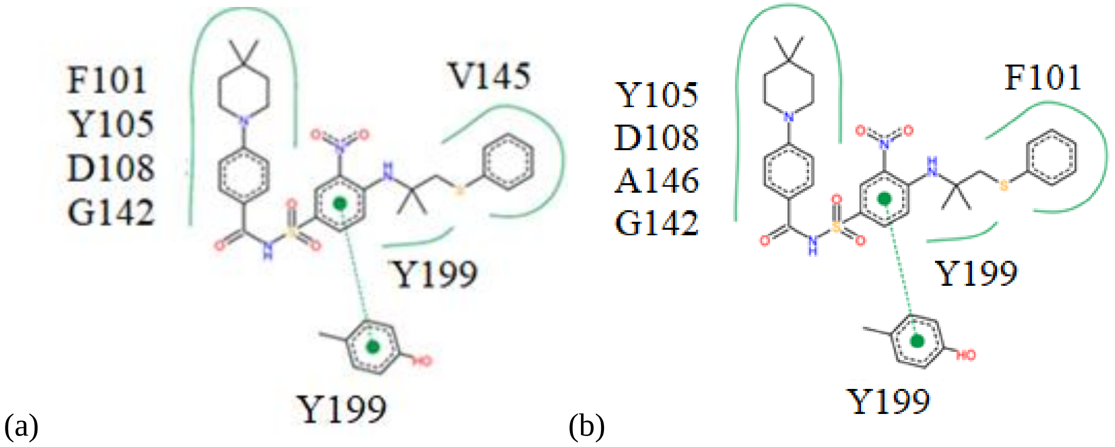

Figure S6. Interactions Predicted by the Poseview Server. (a) crystallographic ligand pose (PDB_ID: 2O22) and (b) ligand pose obtained from re-coupling.

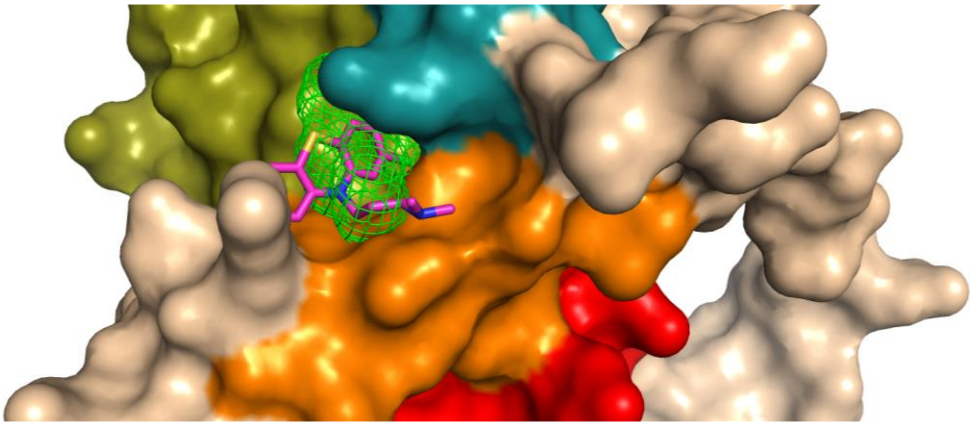

(a)

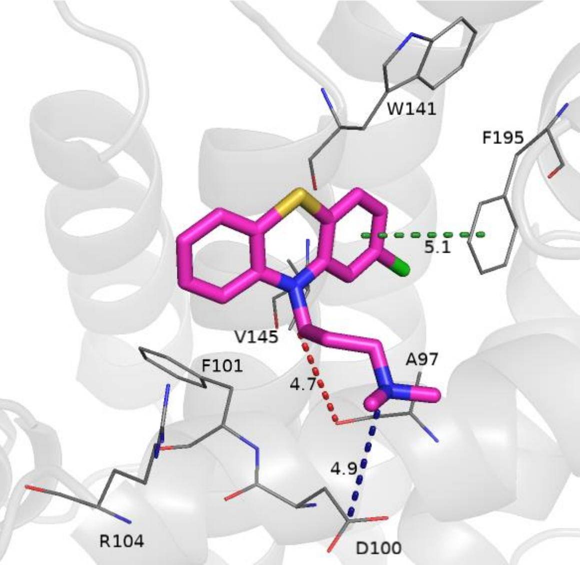

(b)

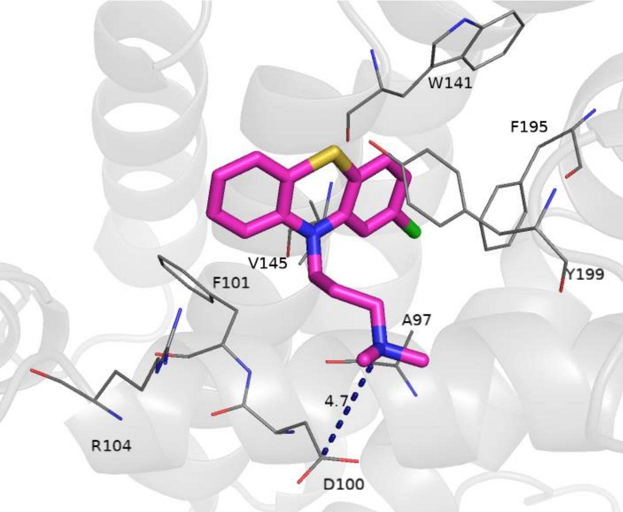

(c)

Figure S7. (a) Representation of BH1-BH4 domains (Figure 2) with presence of site 2 detected by FTSite server with aliphatic chlorpromazine subclass molecular coupling, (EC50 = (125.3 ± 1.1) $\mu mol.L^{-1}$) performed on the AutoDock Vina 1.5.7 program. Representation of the π-stacking (green), hydrogen bonding (red) and saline bridge (Blue) interaction of the molecular couplings performed in AutoDock Vina 1.5.7 (b) and Achilles Blind Docking (c) server, with additional interactions and / or confirmed by the BINANA 1.2.0 algorithm.

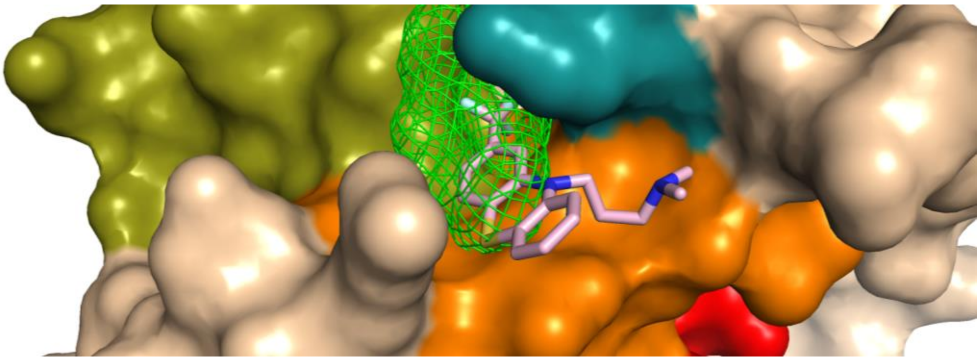

(a)

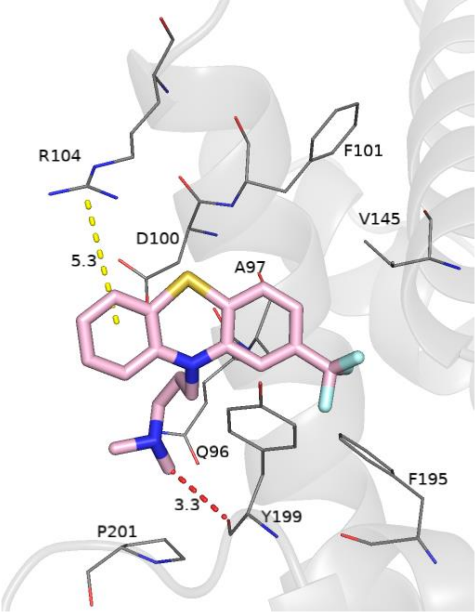

(b)

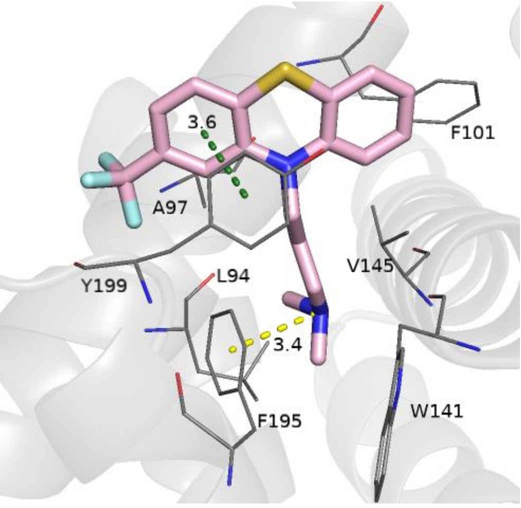

(c)

Figure S8. (a) Representation of BH1-BH4 domains (Figure 2) with the presence of site 2 detected by the FTSite server with the aliphatic subclass triflupromazine molecular coupling (EC50 = (105.9 ± 1.0) μmol.L^{-1}) performed in the AutoDock Vina 1.5.7 program. Representation of π-stacking (green), cation-π (yellow) and hydrogen bonding (red) interactions of the molecular couplings performed in AutoDock Vina 1.5.7 (b) and Achilles Blind Docking (c) server, with interactions additional and / or confirmed by the BINANA 1.2.0 algorithm.

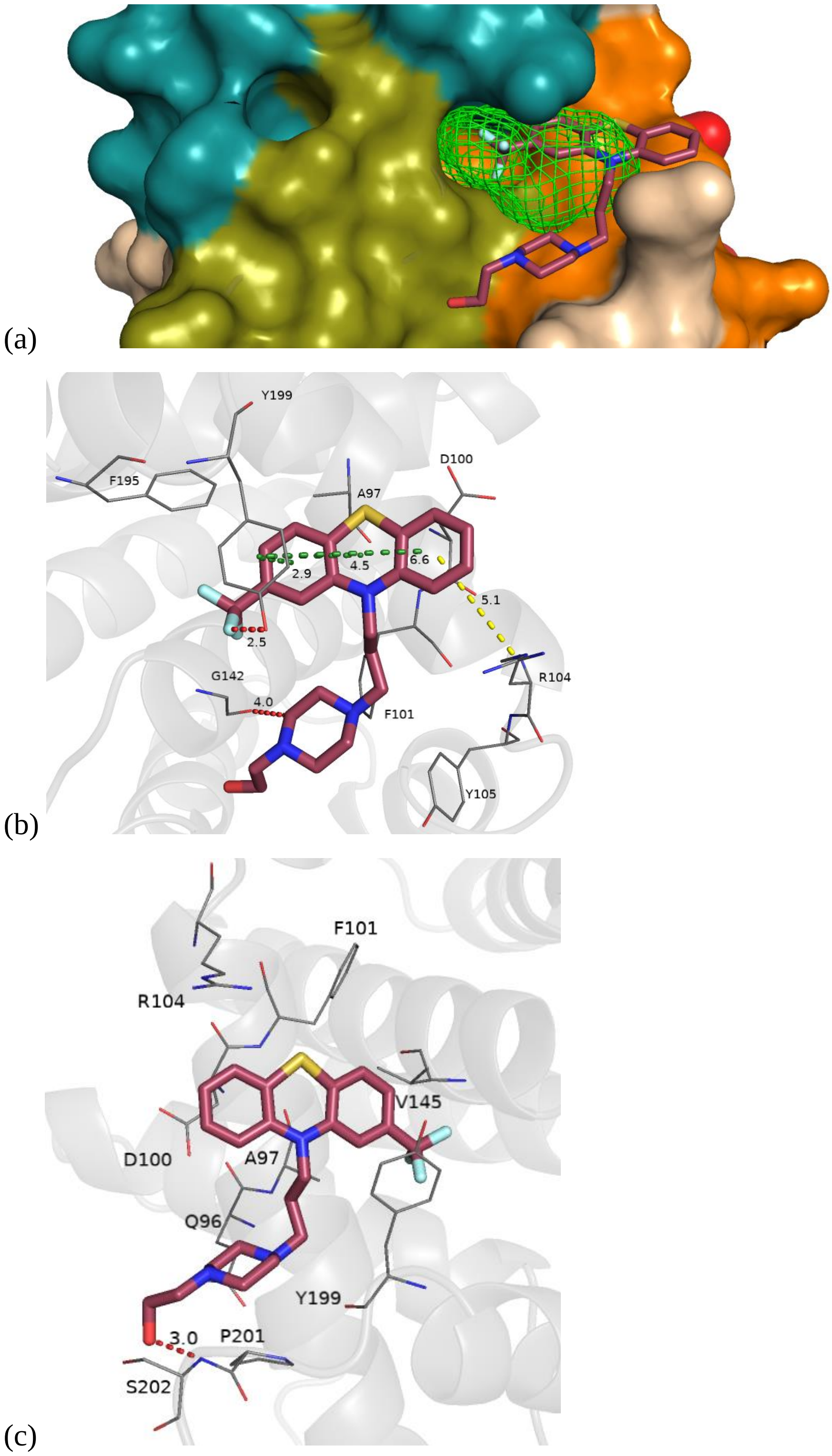

Figure S9. (a) Representation of BH1-BH4 domains (Figure 2) with presence of site 2 detected by FTSite server with piperazine subclass molecular coupling of fluphenazine (EC50 = (63.2 ± 1.0) μmol.L^{-1}) performed in the AutoDock Vina 1.5.7 program. Representation of π-stacking (green), cation-π (yellow) and hydrogen bonding (red) interactions of the molecular couplings performed in AutoDock Vina 1.5.7 (b) and Achilles Blind Docking (c) server, with interactions additional and / or confirmed by the BINANA 1.2.0 algorithm.

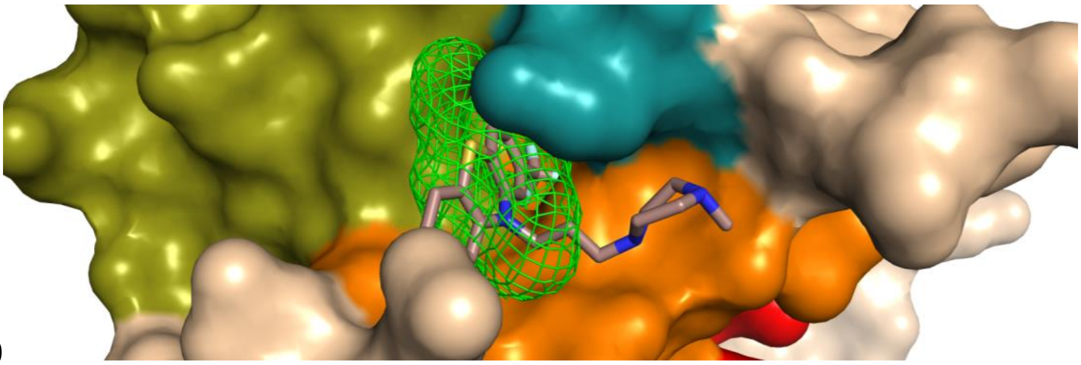

(a)

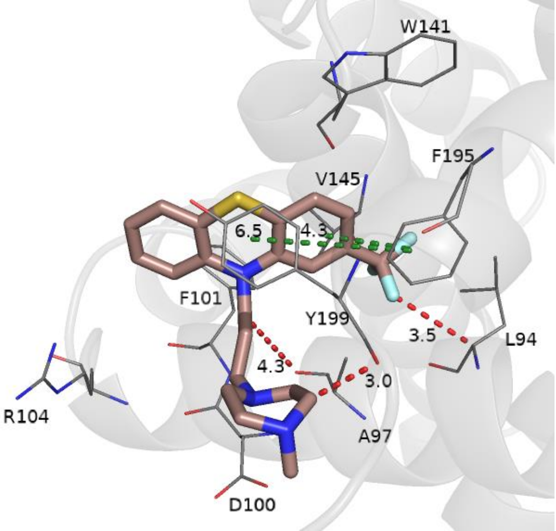

(b)

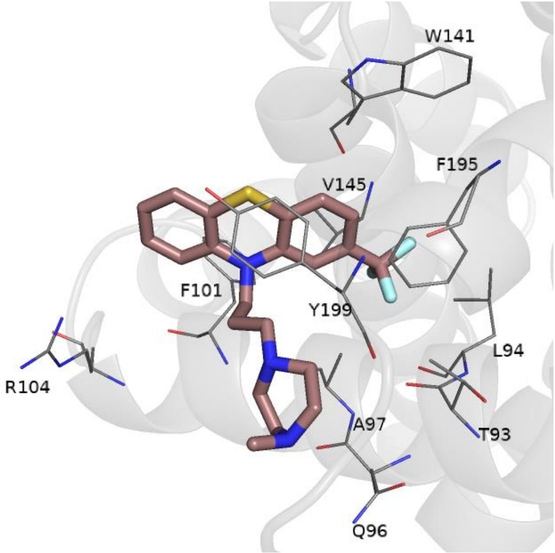

(c)

Figure S10. (a) Representation of BH1-BH4 domains (Figure 2) with the presence of site 2 detected by the FTSite server with the piperazine subclass trifluoperazine molecular coupling (EC50 = (56.2 ± 1.0) μmol.L^{-1}) performed in the AutoDock Vina 1.5.7 program. Representation of the π-stacking (green), hydrogen bonding (red) and saline bridge (Blue) interaction of the molecular couplings performed in AutoDock Vina 1.5.7 (b) and Achilles Blind Docking (c) server, with additional interactions and / or confirmed by the BINANA 1.2.0 algorithm.

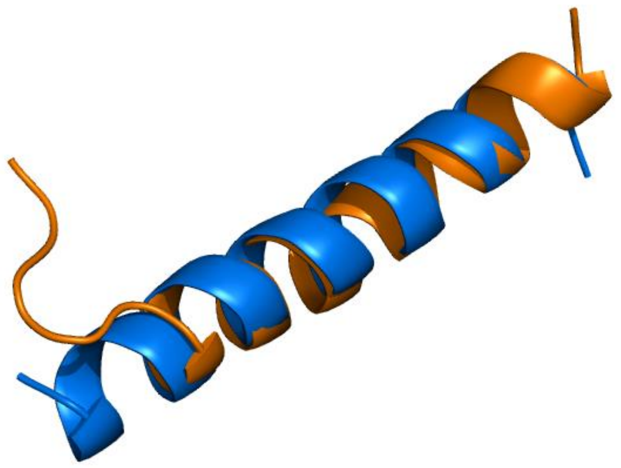

Figure S11. Representation of the molecular coupling of peptide BH3 in the original position of the crystalline structure (blue) (PDB_ID: 2XA0) in relation to the composition used by the coupling (orange) with the GalaxyPepDock server (RMSD 1.962 Å between the alpha helix amino acid residues).

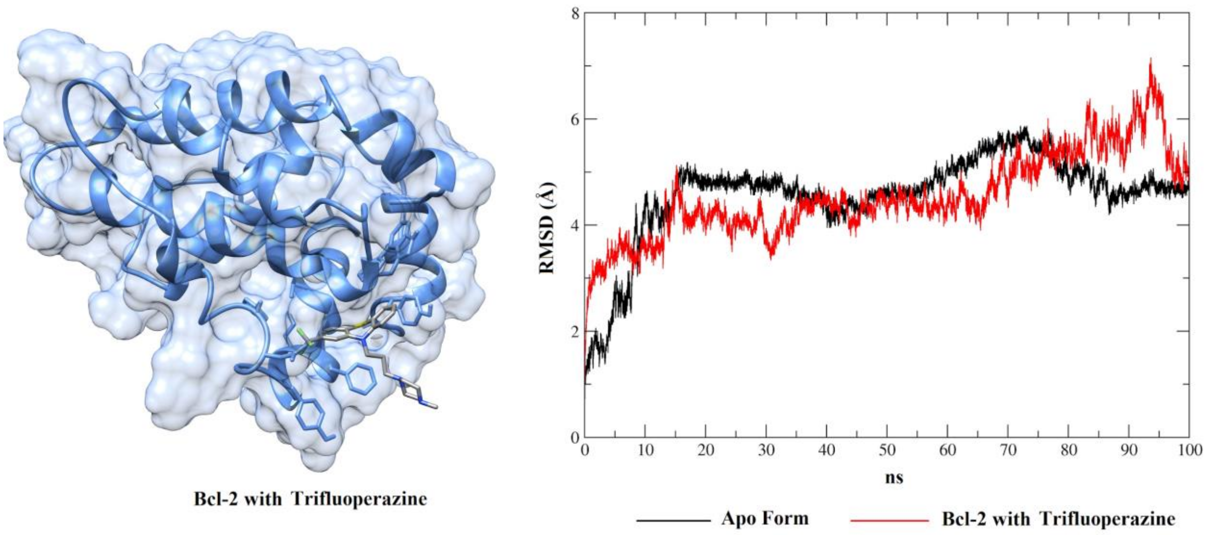

Figure S12. RMSD values of BCL-2 in Apo form (black), BCL-2 with trifluoperazine molecule (red) and 3D conformation obtained by clustering the MD.

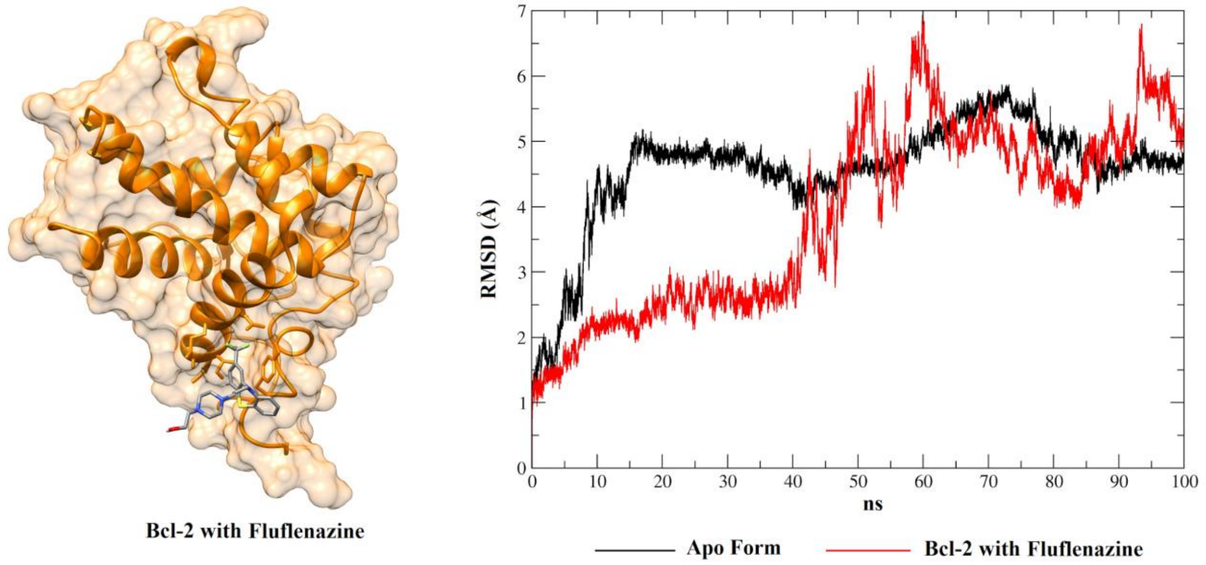

Figure S13. RMSD values of BCL-2 in Apo form (black), BCL-2 with fluphenazine molecule (red) and 3D conformation obtained by clustering the MD.

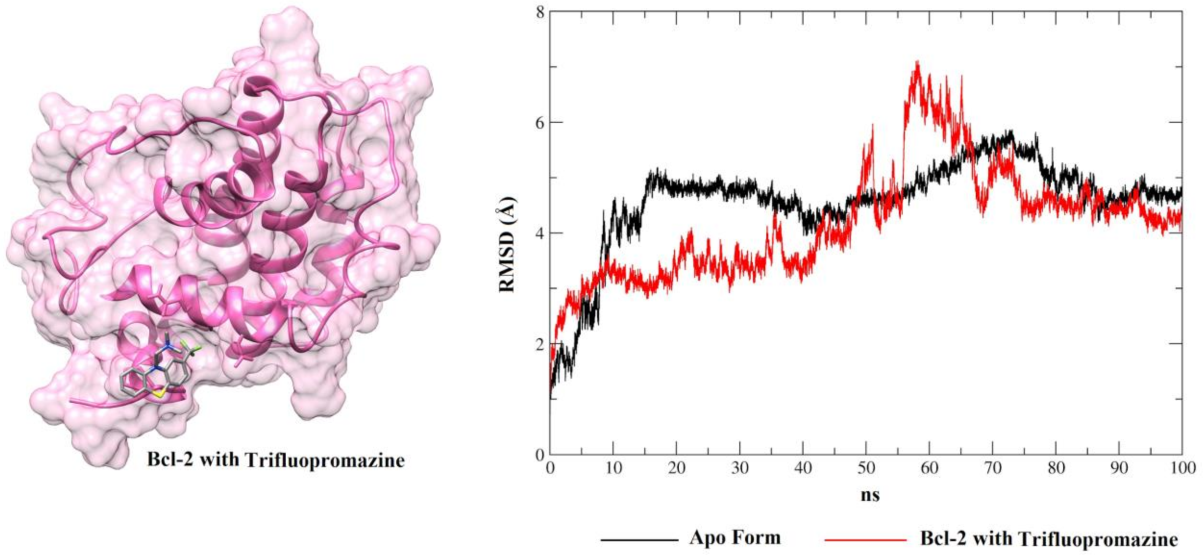

Figure S14. RMSD values of BCL-2 in Apo form (black), BCL-2 with trifluopromazine molecule (red) and 3D conformation obtained by clustering the MD.

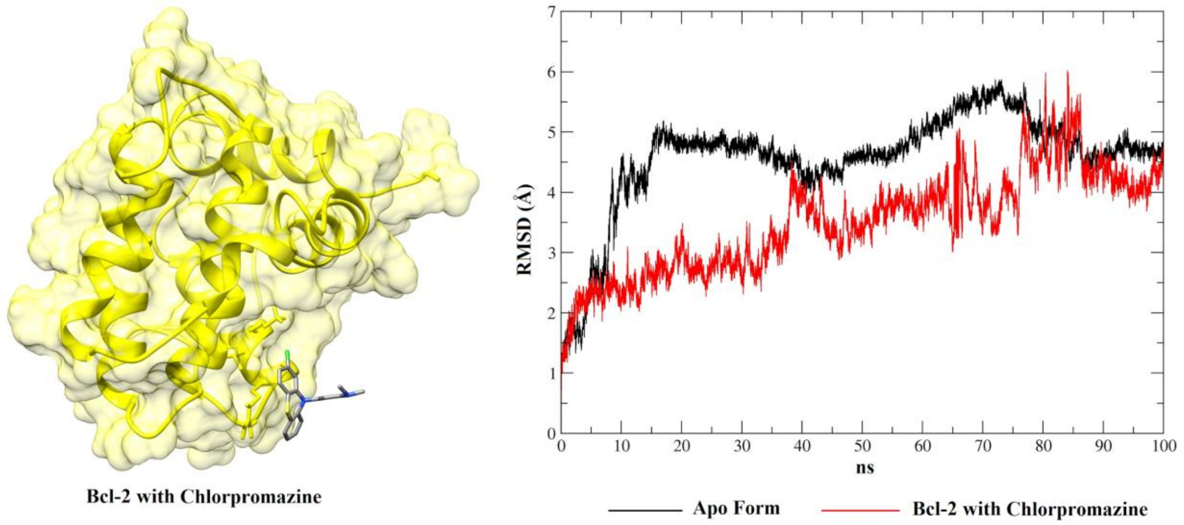

Figure S15. RMSD values of BCL-2 in Apo form (black), BCL-2 with chlorpromazine molecule (red) and 3D conformation obtained by clustering the MD.

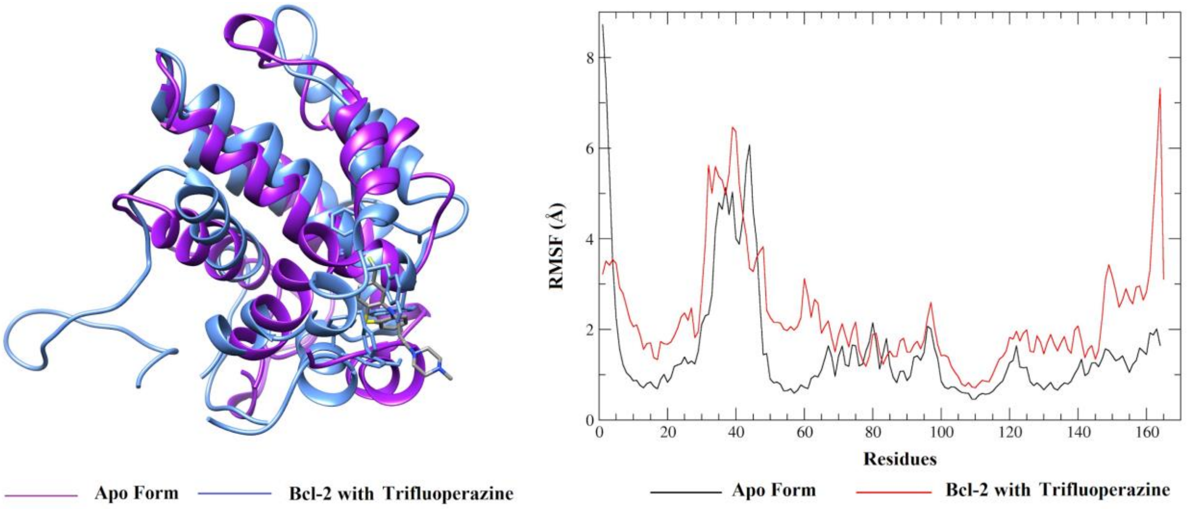

Figure S16. RMSF values of BCL-2 in the presence of trifluoperazine.

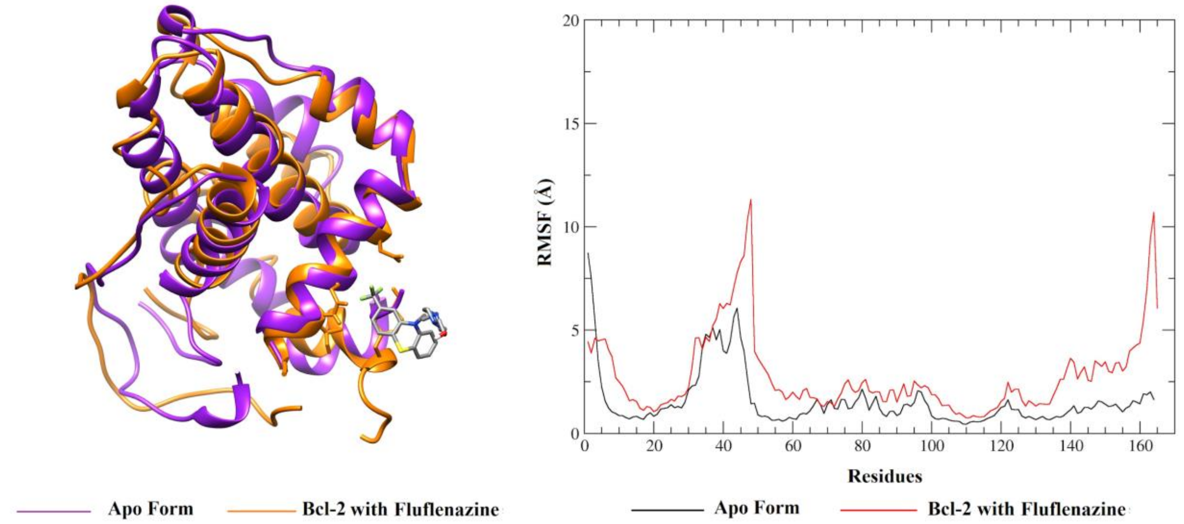

Figure S17. RMSF values of BCL-2 in the presence of fluphenazine.

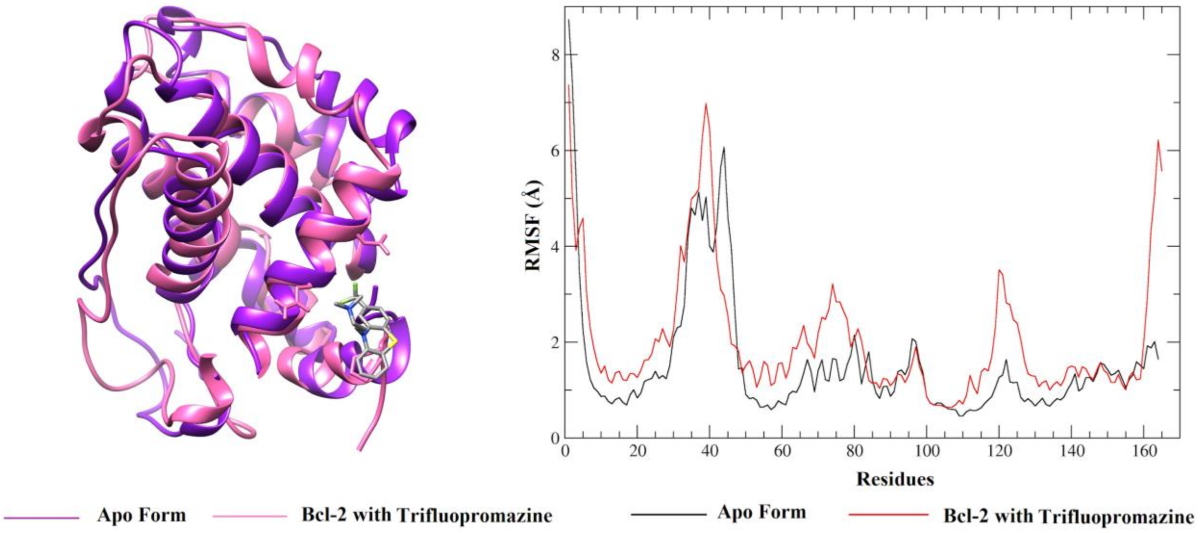

Figure S18. RMSF values of BCL-2 in the presence of triflupromazine.

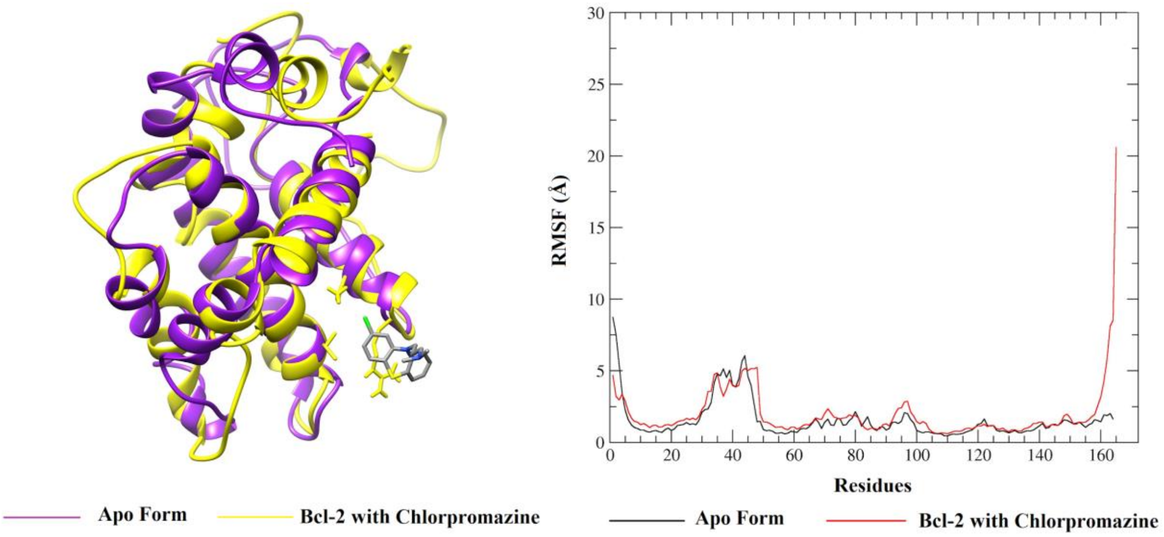

Figure S19. RMSF values of BCL-2 in the presence of chlorpromazine.

REFERENCES

Bohnuud, Tanggis et al. 2012. “Computational Mapping Reveals Dramatic Effect of Hoogsteen Breathing on Duplex DNA Reactivity with Formaldehyde.” *Nucleic Acids Research* 40(16): 7644–52. https://academic.oup.com/nar/article/40/16/7644/1032290.

Brenke, Ryan et al. 2009. “Fragment-Based Identification of Druggable ‘Hot Spots’ of Proteins Using Fourier Domain Correlation Techniques.” *Bioinformatics* 25(5): 621–27. https://academic.oup.com/bioinformatics/article-lookup/doi/10.1093/bioinformatics/btp036.

Kozakov, D. et al. 2011. “Structural Conservation of Druggable Hot Spots in Protein-Protein Interfaces.” *Proceedings of the National Academy of Sciences* 108(33): 13528–33. http://www.pnas.org/cgi/doi/10.1073/pnas.1101835108.

Kozakov, Dima et al. 2015. “The FTMap Family of Web Servers for Determining and Characterizing Ligand-Binding Hot Spots of Proteins.” *Nature Protocols* 10(5): 733–55. http://www.nature.com/articles/nprot.2015.043.

Kyte, Jack, and Russell F. Doolittle. 1982. “A Simple Method for Displaying the Hydropathic Character of a Protein.” *Journal of Molecular Biology* 157(1): 105–32. https://linkinghub.elsevier.com/retrieve/pii/0022283682905150.

Ngan, Chi-Ho et al. 2012. “FTSite: High Accuracy Detection of Ligand Binding Sites on Unbound Protein Structures.” *Bioinformatics* 28(2): 286–87. https://academic.oup.com/bioinformatics/article-lookup/doi/10.1093/bioinformatics/btr651.

De Oliveira Rodrigues, Thiago Assis, Larissa Fernandes Leijôto, Poliane C. Oliveira Brandão, and Cristiane Neri Nobre. 2015. “Predição de Função de Proteínas Através Da Extração de Características Físico-Químicas.” *Revista de Informática Teórica e Aplicada* 22(1): 29. http://seer.ufrgs.br/index.php/rita/article/view/RITA-VOL22-NR1-29.

Pettersen, Eric F. et al. 2004. “UCSF Chimera?A Visualization System for Exploratory Research and Analysis.” *Journal of Computational Chemistry* 25(13): 1605–12. http://doi.wiley.com/10.1002/jcc.20084.
